# Composition and processing activity of a semi-recombinant holo U7 snRNP

**DOI:** 10.1093/nar/gkz1148

**Published:** 2019-12-10

**Authors:** Katarzyna Bucholc, Wei Shen Aik, Xiao-cui Yang, Kaituo Wang, Z Hong Zhou, Michał Dadlez, William F Marzluff, Liang Tong, Zbigniew Dominski

**Affiliations:** 1 Integrative Program for Biological and Genome Sciences, University of North Carolina at Chapel Hill, Chapel Hill, NC 27599, USA; 2 Department of Biophysics, Institute of Biochemistry and Biophysics, Polish Academy of Sciences, 02-106 Warsaw, Poland; 3 Department of Biological Sciences, Columbia University, New York, NY 10027, USA; 4 California NanoSystems Institute, University of California Los Angeles, Los Angeles, CA 90095, USA; 5 Institute of Genetics and Biotechnology, Warsaw University, 02-106 Warsaw, Poland; 6 Department of Biochemistry and Biophysics, University of North Carolina at Chapel Hill, Chapel Hill, NC 27599, USA

## Abstract

In animal cells, replication-dependent histone pre-mRNAs are cleaved at the 3′ end by U7 snRNP consisting of two core components: a ∼60-nucleotide U7 snRNA and a ring of seven proteins, with Lsm10 and Lsm11 replacing the spliceosomal SmD1 and SmD2. Lsm11 interacts with FLASH and together they recruit the endonuclease CPSF73 and other polyadenylation factors, forming catalytically active holo U7 snRNP. Here, we assembled core U7 snRNP bound to FLASH from recombinant components and analyzed its appearance by electron microscopy and ability to support histone pre-mRNA processing in the presence of polyadenylation factors from nuclear extracts. We demonstrate that semi-recombinant holo U7 snRNP reconstituted in this manner has the same composition and functional properties as endogenous U7 snRNP, and accurately cleaves histone pre-mRNAs in a reconstituted *in vitro* processing reaction. We also demonstrate that the U7-specific Sm ring assembles efficiently *in vitro* on a spliceosomal Sm site but the engineered U7 snRNP is functionally impaired. This approach offers a unique opportunity to study the importance of various regions in the Sm proteins and U7 snRNA in 3′ end processing of histone pre-mRNAs.

## INTRODUCTION

Metazoan replication-dependent histone mRNAs are the only known eukaryotic mRNAs that are not polyadenylated, ending instead with a conserved stem-loop followed by a short single-stranded tail of 4–5 nucleotides (1,2). They are formed from longer mRNA precursors (pre-mRNAs) by a single endonucleolytic cleavage carried out by U7 snRNP, a metazoan-specific minor snRNP that is ∼500-fold less abundant than the major spliceosomal snRNPs. Its RNA component, U7 snRNA, is the shortest known snRNA (∼60 nucleotides) and consists of three functionally distinct regions (3–6). The 5′ end region of ∼15 nucleotides base pairs with the sequence in histone pre-mRNA known as Histone Downstream Element (HDE). This region is primarily responsible for the substrate specificity of U7 snRNP for histone pre-mRNAs. The 9-nucleotide AAUUUGUCU sequence located immediately downstream of the 5′ end region is referred to as the Sm binding site (7). This sequence serves as an assembly site for the unique heptameric Sm ring of the U7 snRNP in which Lsm10 and Lsm11 replace the two spliceosomal subunits, SmD1 and SmD2 (8,9). The remaining five subunits, SmE, SmF, SmG, SmB and SmD3, are shared by the two Sm ring types (8,10). The Sm binding site in U7 snRNA is followed by an extensive 3′ stem-loop that *in vivo* may facilitate the assembly of the Sm ring and protect U7 snRNA against the activity of 3′ exonucleases.

Lsm11 is larger than other proteins of the Sm/Lsm family, containing an extended N-terminal region of ∼150 amino acids (9). Residues 20–50 of this region interact with the N-terminal region of FLASH (11) that self-associates into a coiled-coil dimer consisting of two parallel α helices (12). The heterotrimeric Lsm11/FLASH complex functions as a docking platform for a group of four major polyadenylation proteins that we refer to as the Histone pre-mRNA Cleavage Complex (HCC): symplekin, CPSF100, CPSF73 and CstF64 (13–15). The remaining CPSF subunits (CPSF160, WDR33, Fip1 and CPSF30) are detected in the HCC in substoichiometric amounts. These subunits form a module that recognizes the AAUAAA sequence in canonical pre-mRNAs (16–20) and likely represent contaminants of U7 snRNP rather than genuine HCC subunits. Other components of the cleavage and polyadenylation machinery (21,22), including the two remaining CstF subunits, are absent.

The recruitment of the HCC converts U7 snRNP to a catalytically active holo U7 snRNP (14,15). Within the HCC, CPSF73 contacts the pre-mRNA and functions as the endonuclease (23,24). CPSF100 is a homologue of CPSF73 but lacks key residues of the active site (24–26), and symplekin is likely a scaffold that was characterized as a heat sensitive component of the U7 snRNP (27). RNAi studies suggest that CstF64 is not required for the function of U7 snRNP in *Drosophila* (14,28), although it may be essential for 3′ end processing of histone pre-mRNAs in mammalian cells (29,30).

In addition to U7 snRNP, 3′ end processing of histone pre-mRNA requires Stem–Loop Binding Protein (SLBP). SLBP tightly binds the highly conserved stem-loop structure located upstream of the HDE (31–33) and contacts a component of U7 snRNP, likely the FLASH/Lsm11 complex (34), helping to anchor U7 snRNP on histone pre-mRNA. Substrates that form a strong duplex with the U7 snRNA are processed in mammalian nuclear extracts in the absence of SLBP (35–37). Following stable binding of the U7 snRNP to the HDE, histone pre-mRNAs are cleaved by CPSF73 between the stem-loop and the HDE (23,38), with the upstream cleavage product representing mature histone mRNA. The downstream cleavage product containing the HDE is degraded by the 5′-3′ exonuclease activity of CPSF73, releasing the U7 snRNP from the base pair interaction for the next round of processing (23,39).

*In vivo*, the spliceosomal Sm ring is assembled from three pre-formed sub-complexes, SmE/SmF/SmG, SmD1/SmD2 and SmB/SmD3 (40,41), in a multi-step process controlled by the Survival of Motor Neurons (SMN) complex (42–44). The assembly of the U7-specific Sm ring *in vivo* is also controlled by the SMN complex, with the SmD1/SmD2 sub-complex being replaced by the Lsm10/Lsm11 sub-complex (9,45). The assembly of the spliceosomal Sm rings was successfully reproduced *in vitro* in the absence of the SMN complex using either endogenous or recombinant components. These studies generated core U1, U2, U4 and U5 snRNPs suitable for both structural and functional studies (41,46–50).

Here, we used three recombinant sub-complexes, SmE/SmF/SmG, SmB/SmD3 and Lsm10/Lsm11, to assemble the U7-specific Sm ring onto U7 snRNA *in vitro*. The resultant core U7 snRNP was bound to bacterially expressed N-terminal FLASH and incubated with a mouse nuclear extract to recruit the endogenous HCC. Our studies show that the semi-recombinant holo U7 snRNP reconstituted in this manner has the same composition as U7 snRNP purified from nuclear extracts and is active in 3′ end processing of histone pre-mRNAs, functionally mimicking endogenous U7 snRNP. U7 snRNP is limiting in animal cells and its purification in large quantities and native state has proven difficult in the past (10). This report is the first step toward reconstituting holo U7 snRNP entirely from recombinant components for future detailed functional and structural studies.

## MATERIALS AND METHODS

### RNAs

RNAs were generated by T7 transcription from linearized plasmids or double stranded oligonucleotides containing a T7 promoter, or synthesized by GE Dharmacon (Lafayette, CO, USA), as listed below. All sequences are written in 5′-3′ orientation. pc (photo-cleavable linker), m (2′-*O*-methyl modification), 18S (18 atom spacer).

- Human U7 snRNA, GE Dharmacon, 63 nucleotides: CAGUGUUACAGCUCUUUUAGAAUUUGUCUAGUAGGCUUUCUGGCUUUUUACCGGAAAGCCCCU
- Human U7-5′pcB snRNA, GE Dharmacon, 60 nucleotides: 5′Biotin/pc/18S/18S/AGUGUUACAGCUCUUUUAGAAUUUGUCUAGUAGGCUUUCUGGCUUUUUACCGGAAAGCCC
- Mouse U7 snRNA, linearized plasmid, T7 transcription, 76 nucleotides: GGCGAAUUCAAGUGUUACAGCUCUUUUAGAAUUUGUCUAGCAGGUUUUCUGACUUCGGUCGGAAAACCCCUAAGCU
- Mouse U7/Δ4BP snRNA, duplex DNA, T7 transcription, 51 nucleotides: GGUGUUACAGCUCUUUUAGAAUUUGUCUAGCAGGCUGACUUCGGUCGGCCC
- HybU7/Δ4BP snRNA, duplex DNA, T7 transcription, 56 nucleotides: GGCAGAAAAUUUUAUCUCAUUUAGAAUUUGUCUAGCAGGCUGACUUCGGUCGGCCC
- SupU7 snRNA, linearized plasmid, T7 transcription, 76 nucleotides: GGCGAAUUCAAGUGUUACAGGAGAAAUAGAAUUUGUCUAGCAGGUUUUCUGACUUCGGUCGGAAAACCCCUAAGCU
- SupU7/Δ4BP snRNA, duplex DNA, T7 transcription, 51 nucleotides: GGUGUUACAGGAGAAAUAGAAUUUGUCUAGCAGGCUGACUUCGGUCGGCCC
- SupU7/ΔSL+5 snRNA (the inserted AUCUG is underlined), Dharmacon, 39 nucleotides: AGUGUUACAGGAGAAAAUCUGUAGAAUUUGUCUAGCAGG
- U7+4 snRNA (the inserted AUCG is underlined), linearized plasmid, T7 transcription, 80 nucleotides: GGCGAAUUCAAGUGUUACAGCUCUUUAUCGUAGAAUUUGUCUAGCAGGUUUUCUGACUUCGGUCGGAAAACCCCUAAGCU
- SupU7+5N snRNA (the inserted UAGAC is underlined), linearized plasmid, T7 transcription, 81 nucleotides: GGCGAAUUCAAGUGUUACAGGAGAAAUAGACUAGAAUUUGUCUAGCAGGUUUUCUGACUUCGGUCGGAAAACCCCUAAGCU
- SupU7-3 snRNA (the inserted AUA is underlined), linearized plasmid, T7 transcription, 73 nucleotides: GGCGAAUUCAAGUGUUACAGGAGAAGAAUUUGUCUAGCAGGUUUUCUGACUUCGGUCGGAAAACCCCUAAGCU
- *Drosophila* U7 snRNA, linearized plasmid, T7 transcription, 85 nucleotides: GGCGAAUUCAUUGAAAAUUUUUAUUCUCUUUGAAAUUUGUCUUGGUGGGACCCUUUGUCUAGGCAUUGAGUGUUCCCGUUAAGCU
- H2a/Ys pre-mRNA, linearized plasmid, T7 transcription, 107 nucleotides: GGCGAAUUCGAGCUCGGUACCAAAAAGGCUCUUUUCAGAGCCACCCACUGAAUCAGAUUUUCUCCUGUGACACUGUAGCCAAGCCGGAGUAGGCUCGAGUGUAAGCU
- ΔSL/Ys pre-mRNA, linearized plasmid, T7 transcription, 80 nucleotides: GGCGAAUUCAGAGCCACCCACUGAAUCAGAUUUUCUCCUGUGACACUGUAGCCAAGCCGGAGUAGGCUCGAGUGUAAGCU
- DCP/Ys RNA, GE Dharmacon, 30 nucleotides: CUGAAUCAGAUUUUCUCCUGUGACACUGUA
- H2a+4 pre-mRNA (the inserted nucleotides are underlined), linearized plasmid, T7 transcription, 89 nucleotides: GGCGAAUUCGAGCUCGGUACCAAAAAGGCUCUUUUCAGAGCCACCCACUGAAUUAAUUCGAUAAAGAGUUGUGUCACGGUAGCCAAGCU
- H2a+4/Ys pre-mRNA (the inserted nucleotides are underlined), linearized plasmid, T7 transcription, 89 nucleotides: GGCGAAUUCGAGCUCGGUACCAAAAAGGCUCUUUUCAGAGCCACCCACUGAAUUAAUUCGAUUUUCUCUUGUGUCACGGUAGCCAAGCU
- H2a+12 pre-mRNA (the inserted nucleotides are underlined), linearized plasmid, T7 transcription, 97 nucleotides: GGCGAAUUCGAGCUCGGUACCAAAAAGGCUCUUUUCAGAGCCACCCACUGAAUUGACCCACCAAUUCGAUAAAGAGUUGUGUCACGGUAGCCAAGCU
- H2a+12/Ys pre-mRNA (the inserted nucleotides are underlined), linearized plasmid, T7 transcription, 97 nucleotides: GGCGAAUUCGAGCUCGGUACCAAAAAGGCUCUUUUCAGAGCCACCCACUGAAUUGACCCACCAAUUCGAUUUUCUCUUGUGUCACGGUAGCCAAGCU
- H2a WT/Ys pre-mRNA, linearized plasmid, T7 transcription, 85 nucleotides: GGCGAAUUCGAGCUCGGUACCAAAAAGGCUCUUUUCAGAGCCACCCACUGAAUUCGAUUUUCUCUUGUGUCACGGUAGCCAAGCU
- αSupU7, GE Dharmacon, 18 nucleotides: mCmUmAmUmUmUmCmUmCmCmUmGmUmAmAmCmAmC

Other RNAs and oligonucleotides were described: mH2a/5m-5′pcB, mH2a/2m-3′B and dH3/2m-3′B, referred previously to as 5′pcB-mH2a/5m, 3′B-mH2a/2m and 3′B-dH3/2m, respectively) pre-mRNAs (15), SL RNA (51), oligonucleotides complementary to the 5′ end of human/mouse U7 snRNA (34).

### Antibodies

Antibodies against human SLBP, Lsm11 and FLASH were described previously (11,52,53). Antibodies recognizing mouse CPSF100 (A301-581A), CPSF73 (A301-091A) and CstF64 (A301-092A) were from Bethyl Laboratories (Montgomery, TX). Anti-symplekin (610644) was from BD Biosciences. Antibodies against SmB and SmD3 were from Sigma and Bethyl Laboratories (Montgomery, TX), respectively. Anti-MBP was from New England Biolabs and anti-GST was kindly provided by the Strahl laboratory (UNC Chapel Hill). All primary antibodies were used in 1:2000 dilution.

### Cell culture and nuclear extract preparation

Mouse myeloma cells were grown by Cell Culture Company (Minneapolis, MN, USA), shipped on wet ice as a soft pellet and used to prepare nuclear extract the following day, as described (15,54).

### RNA labeling and *in vitro* processing

T7-generated histone pre-mRNAs were treated with calf intestinal phosphatase (New England Biolabs) to remove the 5′ triphosphate and labeled at the 5′ end with ^32^P using T4 polynucleotide kinase (New England Biolabs), as described (37). Synthetic histone pre-mRNAs were labeled at the 5′ end without prior phosphatase treatment. Processing of radioactively labeled histone pre-mRNAs in mouse nuclear extracts was carried out as described (55,56).

### Expression and purification of His–Lsm11/MBP–Lsm10 complex

The construct for N-terminal 6xHis-tagged Lsm11 either containing or lacking the internal loop (residues 211–332) was cloned into the baculovirus pFL vector (57) under the control of a PH promoter. N-terminal maltose binding protein (MBP)-tagged Lsm10 was cloned into the same vector under the control of a P10 promoter. Both proteins were co-expressed in Hi5 insect cells using the baculovirus expression system. Cells were harvested 48 h post infection, collected by centrifugation, re-suspended in lysis buffer (20 mM Tris pH 7.5, 500 mM NaCl, 10 mM imidazole, 5% glycerol, SIGMAFAST™ Protease Inhibitor Cocktail, 10 mM β-mercaptoethanol), and lysed by sonication. Cell lysate was centrifuged at 25 000 × g for 40 min at 4°C and the supernatant incubated with nickel beads for 1 h and loaded onto a gravity flow column (Bio-Rad) for affinity purification. The nickel beads were washed with a buffer containing 20 mM Tris pH 7.5, 500 mM NaCl, 40 mM imidazole, and 10 mM β-mercaptoethanol. The protein was eluted with a buffer containing 20 mM Tris pH 7.5, 500 mM NaCl, 500 mM imidazole, 5% glycerol, and 10 mM β-mercaptoethanol. The eluate was diluted 2.5 times with a buffer containing 20 mM HEPES pH 7.5 and 5 mM DTT before being loaded onto a HiTrap Heparin column (GE Healthcare), and the protein complex was eluted with a salt gradient starting with 80% Buffer A (20 mM HEPES pH 7.5, 5 mM DTT) and 20% Buffer B (20 mM HEPES (pH 7.5), 1 M NaCl, 5 mM DTT) to 100% Buffer B. Relevant fractions were pooled and the protein concentrated and stored at −80°C.

### Expression and purification of His-SmD3/SmB complex

N-terminal 6xHis-tagged SmD3 was cloned into pET28a (Novagen), while untagged full length SmB and SmB encompassing residues 1–95 were cloned into pCDFDuet MCS2. Both genes were co-expressed in *Escherichia coli* BL21 Star (DE3) strain (Novagen) for 18 h at 20°C. Cells were harvested by centrifugation, re-suspended in lysis buffer (20 mM Tris pH 7.5, 500 mM NaCl, 10 mM imidazole, 5% glycerol, 17.8 μg/ml PMSF, 10 mM β-mercaptoethanol) and purified using nickel affinity, as described above. The eluted protein was further purified by heparin affinity using a gradient of Buffer A (20 mM HEPES pH 7.5, 5 mM DTT) and Buffer B (20 mM HEPES pH 7.5, 1 M NaCl, 5 mM DTT) starting with 40% Buffer B to 100% Buffer B. The purified complex was concentrated and stored at −80°C.

### Expression and purification of SmG-His/SmE/SmF complex

C-terminal 6xHis-tagged SmG was cloned into pET26b, while untagged SmE and SmF were cloned into pCDFDuet MCS1 and MCS2, respectively. All three proteins were co-expressed in *E. coli* BL21 Star (DE3) strain (Novagen) and purified as described above for the 6xHis-SmD3/SmB complex.

### Specimen preparation for electron microscopy and data collection

Equimolar amounts of 6xHis-Lsm11(Δ211–332)/MBP-Lsm10, His-SmD3/SmB(1–95), SmG-His/SmE/SmF, U7 snRNA (nucleotides 20–63) and two molar equivalent of FLASH (51–137) C54S/C83A double mutant were mixed in an assembly buffer containing 20 mM HEPES pH 7.5, 250 mM NaCl, 5 mM MgCl_2_ and 5 mM DTT. Prior to mixing, the RNA was heated at 90°C for 5 min and snap cooled on ice for 10 min. The mixture of all the above components was incubated at 30°C for 30 min, followed by an incubation at 37°C for 15 min (58), then cooled on ice for 10 min and incubated overnight at 4°C with TEV protease to remove the MBP tag from Lsm10. The complex was purified by size exclusion chromatography using a Superdex 200 column (GE Healthcare) in the following buffer: 20 mM HEPES pH 7.5, 400 mM NaCl, 1.5 mM MgCl_2_ and 5 mM DTT. Fractions from size exclusion chromatography were analyzed by electrophoresis in 18% SDS/polyacrylamide gels and Coomassie blue staining. Purified samples of the U7 snRNP-FLASH complex were stained using 2% uranyl acetate on a continuous carbon grid and examined by negative stain electron microscopy. Several images were taken using an FEI TF20 transmission electron microscope to determine sample quality. For cryo-electron microscopy, the grids were prepared using an FEI Vitrobot Mark IV set at 4°C and 100% humidity. A sample (3 μl) was applied onto a glow-discharged Quantifoil 300 mesh 1.2/1.3 copper grid, incubated for 10 s and blotted for 5.5 s with a blot force of −3. After blotting, a 0.5 s wait time was applied before the sample was plunge-frozen in liquid ethane. For cryo-EM data, 1135 stacks of images were collected on a Titan Krios equipped with a Volta Phase Plate at the New York Structural Biology Center. The images were recorded with a K2 Summit Camera using the counting mode at a nominal magnification of 29 000× (pixel size = 0.85 Å) at a fixed defocus of −0.33 μm. For each image, a total exposure time of 6 s was recorded in 30 frames with a dose rate of 42 e^–^/Å^2^. The images were motion corrected and dose-weighted using MotionCor2 (59). Initially, 520 499 particles were selected using Gautomatch (www.mrc-lmb.cam.ac.uk/kzhang/Gautomatch/). The particles were windowed into 144 × 144 pixel boxes. CTF parameters were determined using CTFFIND4 (60). Several rounds of 2D classification were performed using Relion 3.0 (61) to select for good quality particles.

### *In vitro* assembly of core U7 snRNP for functional studies

The assembly of core U7 snRNP for functional studies was carried out in a high salt assembly buffer containing 600 mM KCl, 15 mM HEPES pH 7.9, 15% glycerol, 0.25 μg/μl yeast tRNA and 20 mM EDTA. In some experiments, SmB contained only the N-terminal region encompassing amino acids 1–95, and Lsm11 lacked a large loop (amino acids 211–332) that separates Sm motifs 1 and 2. Core U7 snRNP was assembled in a final volume of 100 μl containing 75 μl of the assembly buffer and each component at 20 μM. If necessary, the assembly reaction was appropriately scaled up, maintaining each component at 20 μM. Lsm10/Lsm11 and SmE/SmF/SmG sub-complexes were first mixed in the assembly buffer with U7 snRNA and incubated together at 32°C for 90 min. The assembly reaction was subsequently supplemented with the remaining SmB/SmD3 sub-complex and incubated at 32°C for additional 90 min. The assembled core U7 snRNP was separated from the unbound components by size exclusion chromatography (SEC) using Superose™ 6 Increase 3.2/300 gel filtration column (GE Healthcare) and a buffer compatible with *in vitro* 3′ end processing reaction: 75 mM KCl, 15 mM HEPES–KOH pH 7.9, 10% glycerol and 20 mM EDTA pH 8. In some experiments, core U7 snRNP prior to purifying by SEC was pre-bound to FLASH (40 μM, 2-fold molar excess compared to the core U7 snRNP). When indicated, SEC step was omitted and the core U7 snRNP either alone or pre-bound to FLASH was directly purified by binding to histone pre-mRNA containing biotin at the 5′ or 3′ end, and immobilized on streptavidin beads for subsequent incubation with mouse nuclear extract and reconstitution of semi-recombinant holo U7 snRNP.

### Purification of processing complexes containing semi-recombinant holo U7 snRNP

Core U7 snRNP/FLASH complex (approximately 100 pmol) was mixed in 1 ml of processing buffer (75 mM KCl, 15 mM HEPES–KOH pH 7.9, 15% glycerol and 20 mM EDTA pH 8) with equimolar amounts of SLBP and histone pre-mRNA containing biotin at the 5′ or 3′ end and spun down at 4°C in a microcentrifuge (10 000 × g for 10 min) to remove potential precipitates. The supernatant was loaded over 30–40 μl of streptavidin beads and rotated for 1 h at 4°C to immobilize histone pre-mRNA bound to recombinant core U7 snRNP. The beads were gently spun down in a microcentrifuge (3 min at 30 × g), rinsed three times with processing buffer and rotated with 1 ml of the same buffer for 1 h and next transferred to a new tube for an additional 1 h rotation with a fresh change of buffer. The beads were subsequently incubated with 1 ml of a mouse nuclear extract that prior to using was supplemented with 80 mM EDTA to a final concentration of 20 mM and spun down at 4°C in a microcentrifuge (10 000 × g for 10 min) to remove precipitates. Following 1 h rotation at 4°C with the pre-cleared nuclear extract, the beads containing immobilized complexes of histone pre-mRNA and semi-recombinant holo U7 snRNP were gently spun down and exhaustively washed, including one tube change, as described above. Processing complexes assembled on mH2a/2m-3′B pre-mRNA were eluted to solution by boiling in SDS sample buffer, separated by electrophoresis in a 4–12% SDS/polyacrylamide gel and analyzed by silver staining and/or western blotting. Processing complexes assembled on mH2a/5m-5′pcB pre-mRNA were UV eluted in 50 μl of processing buffer and were analyzed by Western blotting, silver staining and/or mass spectrometry, as described (15,62).

### Purification of semi-recombinant holo U7 snRNP assembled on U7-5′pcB snRNA

A complex (∼100 pmol) of core U7 snRNP assembled on U7–5′pcB snRNA and bound to FLASH was added to 1 ml of processing buffer (see above) and directly immobilized on 30–40 μl of streptavidin beads and extensively washed with the same buffer, as described above. The beads were rotated with 1 ml of a pre-cleared mouse nuclear extract containing 20 mM EDTA to reconstitute holo U7 snRNP. Following a 1 h rotation with the extract at 4°C, the beads were collected by a gentle spin in a microcentrifuge (3 min at 30 × g) and extensively washed with processing buffer, as described above. Semi-recombinant holo U7 snRNP was released to solution by irradiating a suspension of the beads in processing buffer (75 μl) with long wave UV, as described (15,62). To determine the composition of semi-recombinant holo U7 snRNP, a fraction of the UV eluted solution (10%) was analyzed by Western blotting, silver staining and/or mass spectrometry. Processing activity of semi-recombinant holo U7 snRNP was tested using 0.025 pmol (0.5 ng) of 5′-labeled pre-mRNA substrate. Varying amounts of the UV eluted solution (0.5–5%) were added with or without 10 pmol of SLBP to 7.5 μl of the assembly buffer containing pre-mRNA substrate and 0.25 μg/μl yeast tRNA (Invitrogen). The reaction samples were incubated 60 min at 32°C, treated with proteinase K for 1 h and mixed with 4 volumes of 8 M urea dye. Radioactive RNAs were separated by electrophoresis in 8% polyacrylamide/7 M urea denaturing gels and detected by autoradiography.

### Cleavage activity of complexes immobilized on streptavidin beads

mH2a/2m-3′B pre-mRNA labeled at the 5′ end with ^32^P (∼100 ng or 5 pmol, 50 000 counts per minute) was mixed in 1 ml of processing buffer with 10 pmol of FLAG-tagged full length SLBP and 100 pmol of core U7 snRNP bound to either N139 or Δ61 FLASH. Following a 5 min incubation on ice, the samples were spun down in a microcentrifuge (10 min at 10 000 × g) to remove potential precipitates and the supernatant was rotated with 30–40 μl of streptavidin beads for 1 h at 4°C to bind processing complexes via biotin attached to the 3′ end of mH2a/2m-3′B pre-mRNA. The beads were gently spun down (3 min at 30 × g), washed by rotating with processing buffer for 1 h at 4°C, transferred to a fresh tube and rotated in the same buffer for an additional hour. Mouse nuclear extract (250 μl) containing 20 mM EDTA was mixed with 750 μl of processing buffer, cleared by spinning in a microcentrifuge (10 min at 10 000 × g) and loaded over the extensively washed streptavidin beads containing immobilized complexes. The samples were rotated for 1 h at 4°C to reconstitute holo U7 snRNP, gently spun down and extensively washed using two changes of processing buffer, as described above. One half of the beads was re-suspended in SDS sample buffer and the bound proteins separated by electrophoresis in a 4–12% SDS/polyacrylamide gel and analyzed by western blotting or silver staining. The remaining half of the beads was re-suspended in processing buffer, divided in small aliquots and left on ice or rotated at 32°C for 90 min to evaluate processing activity. The RNA was recovered from the beads by boiling for 5 min in the presence of 2% SDS and 0.3 M sodium acetate, phenol extraction and ethanol precipitation. The precipitated RNA was separated by gel electrophoresis in 8%/7 M urea gel and detected by autoradiography.

### Direct analysis of processing activity of recombinant core U7 snRNPs in nuclear extracts

Recombinant core U7 snRNPs assembled on U7 snRNA with wild type mouse/human 5′ region (thus having the same substrate specificity as endogenous mouse U7 snRNA) prior to adding to a mouse nuclear extract were bound to histone pre-mRNA. This pre-binding step minimized the contribution of endogenous U7 snRNP to processing. Approximately 1 pmol of recombinant core U7 snRNP was mixed with 7.5 μl of processing buffer and 0.025 pmol of radioactive substrate, incubated on ice for 5 min and the complex added to 7.5 μl of a mouse nuclear extract containing 20 mM EDTA. Our titration experiments demonstrated that this amount of nuclear extract contains sufficient amounts of free HCC to assemble 1 pmol of core U7 snRNP into fully active semi-recombinant holo U7 snRNP. Recombinant core U7 snRNPs assembled on U7 snRNA that contained altered 5′ region, including SupU7, HybU7 and DmU7 (thus having different substrate specificity than endogenous mouse U7 snRNA), were directly added to the nuclear extract and tested for processing activity with their respective substrates. The reaction samples were incubated for 60 min at 32°C and the radioactive RNA was analyzed, as described above.

## RESULTS

### Assembly and electron microscopy studies of the core U7 snRNP/FLASH complex

The seven proteins of the U7 ring were expressed as three sub-complexes. His-Lsm11(Δ211–332) and MBP-Lsm10 were expressed in baculovirus-infected insect cells from a single vector under the control of two separate promoters. Lsm11 lacked amino acids 211–332 that encompass a large loop between Sm1 and Sm2 motifs, and Lsm10 was full length and fused to maltose binding protein (MBP) to enhance solubility. Expression of this complex in *E. coli* was unsuccessful. An SmG-His/SmE/SmF ternary complex and a His-SmD3/SmB(1–95) binary complex were expressed in *E. coli*. SmB lacked the unstructured C-terminal region located between amino acids 96 and 231 that was likely to affect alignment of the U7 snRNP particles in electron microscopy.

**Figure 1.**
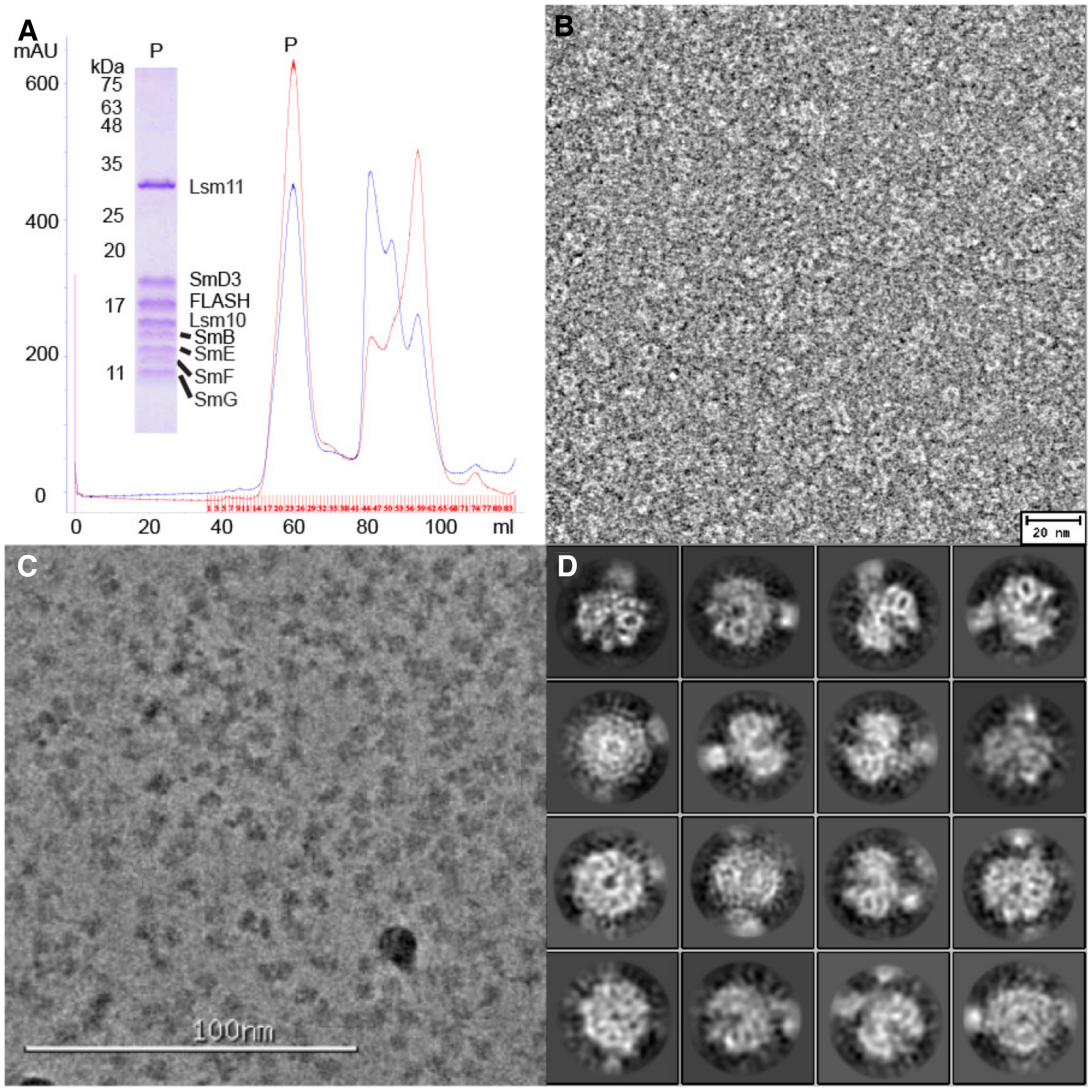
Reconstitution and EM studies of core U7 snRNP. (**A**) Gel filtration profile of assembled core U7 snRNP in a complex with FLASH. The complex elutes in the first sharp and nearly symmetrical peak (P). A Coomassie blue-stained SDS-PAGE gel of the complex is shown in the insert. (**B**) Negative-stain image of the ring-shaped structures of the core U7 snRNP-FLASH complex. (**C**) Cryo-EM image of the core U7 snRNP-FLASH complex. (**D**) 2D classification of the particles in the cryo-EM images. Density for the U7 snRNP Sm ring and possibly the stem-loop of the U7 RNA could be recognized. 39 270 particles were selected from the images and classified.

To generate the core U7 snRNP/FLASH complex for electron microscopy studies, the three sub-complexes were mixed together with a truncated version of human U7 snRNA (nucleotides 20–63) and the C54S/C83A double mutant of the N-terminal fragment of FLASH (amino acids 51–137) expressed in *E. coli* (12). The MBP tag was removed by proteolysis, and the complex was purified by size exclusion chromatography (SEC), eluting as a single peak from the column (Figure 1A). SDS-PAGE analysis of the peak fraction showed that it contained all eight proteins of the core U7 snRNP/FLASH complex (Figure 1A).

We first carried out negative-stain electron microscopy (EM) to assess the quality and suitability of the purified U7 snRNP/FLASH complex for cryo-electron microscopy (cryo-EM) studies. Negative-stain images of the complex showed the presence of ring-shaped particles with a diameter of ∼80 Å (Figure 1B), resembling the shape of core U4 snRNP that shares five out of seven Sm subunits with U7 snRNP (48). To obtain a higher resolution visualization of the U7 snRNP/FLASH complex, we carried out cryo-EM studies (Figure 1C) using a Volta phase plate because of the relatively small size of the particles. 2D class averaging yielded objects resembling a donut with an attached tail (Figure 1D), which based on the U4 snRNP structure likely represent the Sm ring of the U7 snRNP and the stem-loop of the U7 snRNA, respectively. However, the small size of the particles and the pseudo-symmetry of the Sm ring precluded us from locating FLASH and obtaining a meaningful 3D reconstruction.

### Composition of semi-recombinant holo U7 snRNP

**Figure 2.**
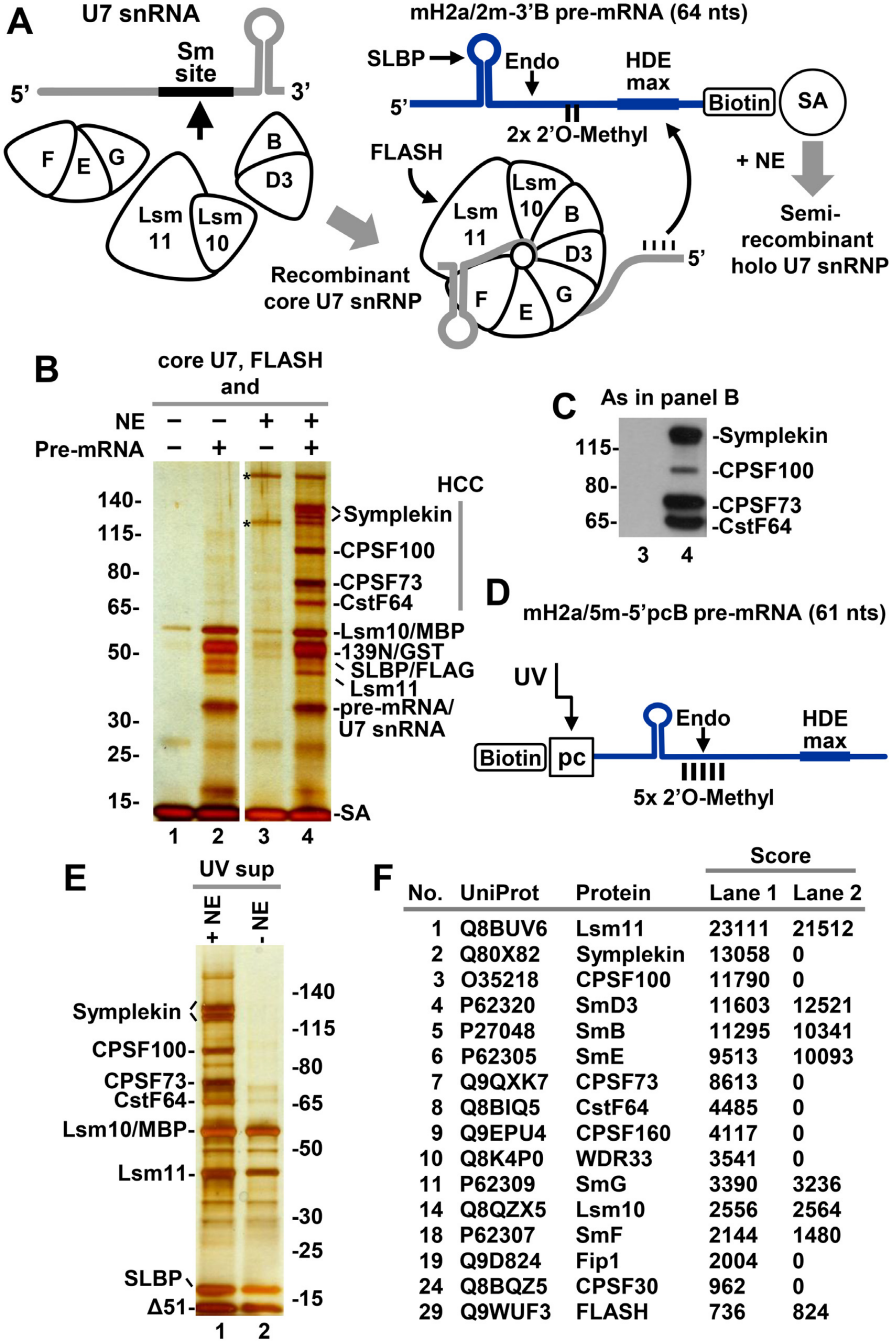
Purification of semi-recombinant holo U7 snRNP via biotin-tagged histone pre-mRNA. (**A**) The experimental outline. The seven proteins of U7-specific ring were expressed as three sub-complexes and bound, depending on experiment, to either synthetic or T7-generated U7 snRNA. The *in vitro* assembled core U7 snRNP was incubated with N-terminal FLASH and the resultant complex bound to mH2a/2m-3′B histone pre-mRNA (or another biotin-tagged pre-mRNA) and immobilized on streptavidin beads (SA) for subsequent incubation with nuclear extract and reconstitution of semi-recombinant holo U7 snRNP. (**B**, **C**) Analysis of immobilized holo U7 snRNP by silver staining and Western blotting. Core U7 snRNP bound to 139N FLASH (amino acids 1–139 N-terminally tagged with GST) was incubated with SA beads in the absence or presence of mH2a/2m-3′B histone pre-mRNA and/or mouse nuclear extract. Immobilized proteins and RNAs were separated by 4–12% SDS/polyacrylamide gel electrophoresis and visualized by silver staining (panel B) or Western blotting (panel C). Note that only lanes 3 and 4 from panel B are used for Western blotting. Asterisks in panel B indicate proteins of the nuclear extract that nonspecifically bind to SA beads. (**D**–**F**). Semi-recombinant holo U7 snRNP was immobilized on SA beads as described in panels A and B with the exception that the recombinant core U7 snRNP was complexed with Δ51 FLASH (lacking the first 51 amino acids that are not required for processing and a GST tag) and bound to mH2a/5m-5′pcB pre-mRNA (panel D) instead of mH2a/2m-3′B. Formation of the complex was facilitated by addition of recombinant SLBP (amino acids 125–223). The immobilized complex was UV eluted and a fraction of solution analyzed by silver staining (panel E) or mass spectrometry (panel F). Lane 2 contains recombinant core U7 snRNP that was bound to mH2a/5m-5′pcB pre-mRNA but not incubated with the mouse nuclear extract.

For functional studies, core U7 snRNP was assembled from the three sub-complexes described above. Lsm11 was either full length or lacked amino acids 211–332. In most experiments, SmB contained only the first 95 amino acids. The MBP tag fused to Lsm10 did not interfere with the processing activity of U7 snRNP (data not shown) and was not removed by proteolysis. The remaining proteins of the Sm ring were full length. Initially, for the assembly of core U7 snRNP we used chemically synthesized 63-nucleotide human U7 snRNA (Figure 2A). The three sub-complexes were mixed in a high salt buffer with equimolar amounts of the synthetic U7 snRNA and the assembled core U7 snRNP was bound to the N-terminal fragment of FLASH via its interaction with Lsm11 and purified by SEC. Depending on the experiment, we used FLASH encompassing amino acids 1–139 (139N) or its truncated forms lacking the first 51 (Δ51) or 61 (Δ61) residues, with each protein being N-terminally fused to either a 6xHis or GST tag. The *in vitro* assembled and purified complex of core U7 snRNP and FLASH was next incubated with a mouse nuclear extract to bind endogenous HCC and evaluated for processing activity using appropriate histone pre-mRNA substrates.

We first tested whether an *in vitro* assembled complex of core U7 snRNP (full length Lsm11 and SmB 1–95) and GST tagged 139N FLASH bound to histone pre-mRNA is capable of recruiting the HCC. An aliquot of the core U7 snRNP/FLASH (50 pmol) was bound in the presence of full length SLBP/FLAG (50 pmol) to 25 pmol of mH2a/2m-3′B pre-mRNA that contained biotin at the 3′ end (Figure 2A) (15,34). The molar excess of core U7 snRNP/FLASH and SLBP served to convert most of the substrate into a complex. The interaction of mH2a/2m-3′B and U7 snRNP is primarily mediated by formation of a strong 16-base pair duplex between the HDE of the pre-mRNA and the 5′ end of the U7 snRNA, and SLBP was added to additionally stabilize this interaction (34). In some experiments, full length SLBP was replaced by a His-tagged truncated version (amino acids 125–223) that retains normal function in processing (34).

The complex containing mH2a/2m-3′B pre-mRNA, SLBP and the *in vitro* assembled core U7 snRNP bound to 139N FLASH was immobilized on streptavidin (SA) beads and incubated on ice with either a buffer, as a control, or a mouse nuclear extract to recruit missing polyadenylation factors of the HCC, hence reconstituting a semi-recombinant holo U7 snRNP. Note that mH2a/2m-3′B pre-mRNA contains two 2′-*O*-methyl groups immediately upstream of the HDE that do not block cleavage but prevent subsequent degradation of the downstream product and displacement of U7 snRNP from the HDE (Figure 2A) (15,34). Following exhaustive washes, components of the immobilized complexes were separated on a 4–12% SDS/polyacrylamide gel and visualized by silver staining. In both samples, GST-tagged 139N FLASH was readily detectable, as were the two largest subunits of the recombinant core U7 snRNP: full length Lsm11, and Lsm10 fused to MBP (Figure 2B, lanes 2 and 4). The complex incubated in the presence of the nuclear extract (Figure 2B, lane 4), but not in the presence of the buffer (Figure 2B, lane 2), additionally contained a number of bands identified by Western blotting as subunits of the HCC: symplekin, CPSF100, CPSF73 and CstF64 (Figure 2C). We conclude that the reconstituted semi-recombinant holo U7 snRNP resembles in its composition endogenous holo U7 snRNP isolated from mouse nuclear extracts using the same approach (34). Only trace amounts of the core U7 snRNP components and two nonspecific proteins of the extract were bound to SA beads in the absence of the pre-mRNA (Figure 2B, lanes 1 and 3). Note that mH2a/2m-3′B pre-mRNA and the *in vitro* assembled core U7 snRNP were pre-bound together prior to incubation with the extract, preventing purification of endogenous U7 snRNP by the pre-mRNA.

We next assembled and purified semi-recombinant holo U7 snRNP using mH2a/5m-5′pcB pre-mRNA rather than mH2a/2m-3′B pre-mRNA (Figure 2D). This substrate contains biotin at the 5′ end followed by a photo-sensitive linker that facilitates selective elution of the immobilized complexes from SA beads by irradiation with long wave UV (366 nm) (15,62). This method yields processing complexes that lack background proteins nonspecifically bound to SA beads and are suitable for direct and unbiased mass spectrometry analysis. The mH2a/5m-5′pcB substrate contains five 2′-*O*-methyl groups around the cleavage site that prevent processing in nuclear extracts, hence increasing the yield of complete complexes (15). Analysis of a small fraction of the UV-eluted complexes by silver staining (Figure 2E), Western blotting (not shown) and mass spectrometry (Figure 2F) further confirmed that the semi-recombinant holo U7 snRNP does not differ in composition from the endogenous holo U7 snRNP, being associated with symplekin, CPSF100, CPSF73 and CstF64. In the UV-eluted samples, mass spectrometry also identified the remaining components of CPSF (CPSF160, WDR33, Fip1 and CPSF30) but silver staining and mass spectrometry suggested they were present in smaller amounts, as previously demonstrated for endogenous U7 snRNP (15,62). We conclude that *in vitro* assembled core U7 snRNP bound to FLASH associates in nuclear extracts with the same subset of polyadenylation factors as the endogenous U7 snRNP.

### Processing activity of purified semi-recombinant holo U7 snRNP

**Figure 3.**
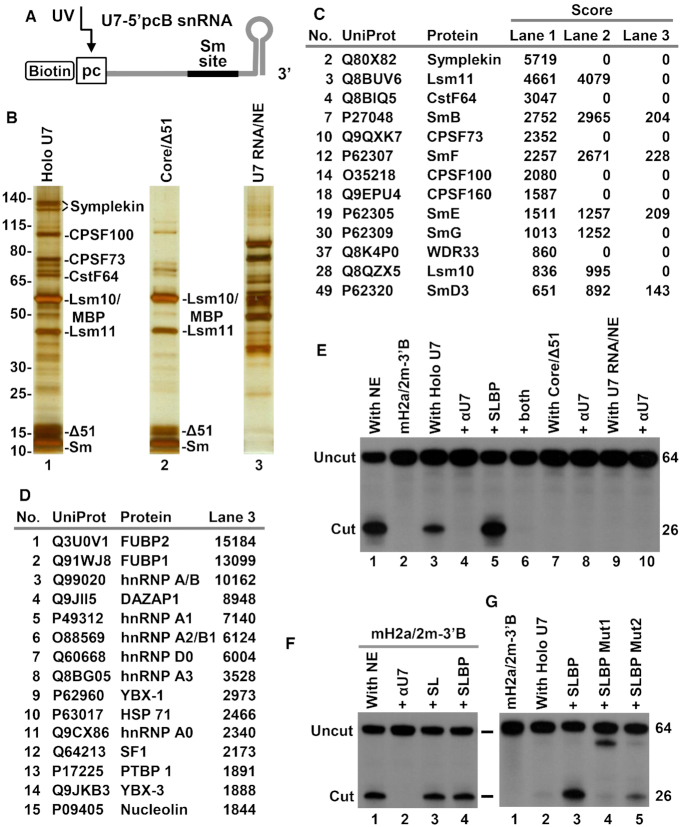
Processing activity of purified semi-recombinant holo U7 snRNP. (**A**) Recombinant core U7 snRNP containing full length Lsm11 and SmB/Δ1–95 was assembled on synthetic U7–5′pcB snRNA tagged at the 5′ end with biotin and a photo-cleavable linker. (**B**) The assembled core was bound to Δ51 FLASH, immobilized on SA beads and incubated with mouse nuclear extract to recruit the HCC. The reconstituted semi-recombinant holo U7 snRNP was eluted to solution by UV irradiation and a fraction (10%) was analyzed by silver staining (lane 1). Silver staining of UV-eluted core U7 snRNP/Δ51 FLASH complex (no incubation with the nuclear extract) is shown in lane 2. Silver staining of UV-eluted U7–5′pcB snRNA (no Sm ring and FLASH) incubated with the same nuclear extract is shown in lane 3. (**C**) The same fraction (10%) of the three UV-eluted samples was analyzed by mass spectrometry. The table lists top scoring components (scores > 500) of semi-recombinant holo U7 snRNP (lane 1 in panel B). (**D**) Top scoring proteins identified in the UV-eluted sample of U7–5′pcB snRNA incubated with the nuclear extract (lane 3 in panel B). (**E**) A fraction (5%) of the UV-eluted samples shown in panel B were tested for the ability to support processing of mH2a/2m-3′B pre-mRNA either in the absence or in the presence of SLBP and/or an oligonucleotide blocking mouse U7 snRNA (αU7), as indicated at the top of each lane. (**F**) Processing of mH2a/2m-3′B pre-mRNA in a mouse nuclear extract alone (lane 1) or in the presence of SLBP, stem–loop RNA (SL) or αU7 oligonucleotide, as indicated at the top of each lane. (**G**) Processing of mH2a/2m-3′B pre-mRNA by a fraction (0.5%) of the UV-eluted semi-recombinant holo U7 snRNP alone (lane 2) or in the presence of WT SLBP (lane 3), or its mutant versions unable to support processing (lanes 4–5).

We took advantage of the UV-elution method to directly purify semi-recombinant holo U7 snRNP on SA beads. The seven recombinant Sm proteins (full length Lsm11, SmB 1–95) and Δ51 FLASH (amino acids 52–139, see also Figure 5A) were bound to chemically synthesized U7–5′pcB snRNA that contains biotin and a photo-cleavable linker at the 5′ end (Figure 3A). The resultant complex was immobilized on SA beads and incubated with the nuclear extract to recruit the HCC. The reconstituted semi-recombinant holo U7 snRNP was extensively washed on the beads and eluted into a buffer by exposure to long wave UV (366 nm). In parallel to purifying the semi-recombinant holo U7 snRNP, we also UV-eluted two additional samples as controls: U7-5′pcB snRNA (no recombinant Sm ring) that was incubated with the same nuclear extract, and core U7 snRNP bound to Δ51 FLASH (no incubation with the extract).

The three UV-eluted samples were analyzed by silver staining (Figure 3B) and mass spectrometry (Figure 3C and D) to determine their composition. Based on both assays, semi-recombinant holo U7 snRNP (Figure 3A, lane 1) contained all recombinant subunits of the Sm ring, Δ51 FLASH, endogenous HCC (symplekin, CPSF100, CPSF73, CstF64) and the remaining CPSF subunits (CPSF160, WDR33, Fip1, CPSF30). In addition, smaller amounts of various RNA binding proteins were also detected. The same RNA binding proteins were much more prevalent in the UV-eluted sample prepared by incubating naked U7-5′pcB snRNA (lacking recombinant Sm ring) in the nuclear extract (Figure 3B, lane 3, Figure 3D). Thus, the presence of a Sm ring prevents U7 snRNA from nonspecifically interacting with abundant RNA-binding proteins of the nuclear extract. The HCC subunits, Lsm11 and Lsm10 were not present, and SmB, SmF, SmE and SmD3 were only detected as background contaminants likely as a result of nonspecific binding of the abundant spliceosomal snRNPs to the beads. The UV-eluted core U7 snRNP that was not incubated with the nuclear extract contained only recombinant proteins used for the assembly: FLASH and the seven Sm subunits (Figure 3B, lane 2, and Figure 3C).

A fraction of each UV-eluted solution (5%) was tested for the ability to cleave 5′ labeled mH2a/2m-3′B pre-mRNA (Figure 2A) under standard processing conditions that include 20 mM EDTA. Note that the UV-eluted samples were free of endogenous U7 snRNP (which lacks the biotin tag) and the pre-mRNA substrate was not exposed at any point to the nuclear extract. Of the three UV-eluted samples, only semi-recombinant holo U7 snRNP cleaved mH2a/2m-3′B pre-mRNA (Figure 3E, lane 3), and as expected core U7 snRNP and U7 snRNA bound to various hnRNPs and other RNA binding proteins of the nuclear extract were inactive (Figure 3E, lanes 7 and 9, respectively). Cleavage mediated by purified semi-recombinant holo U7 snRNP occurred at the same site that is selected by endogenous U7 snRNP (Figure 3E, lane 1) and was blocked by αMmU7, an antisense oligonucleotide complementary to the 5′ end of U7 snRNA (Figure 3E, lane 4). Based on these results we conclude that the purified semi-recombinant U7 snRNP contains all components essential for the catalytic activity. This is the first example of a U7-dependent processing reaction reconstituted from purified components and carried out in a buffer rather than in a protein- and RNA-rich environment of the nuclear extract.

Cleavage was strongly stimulated in the presence of recombinant SLBP (Figure 3E, lane 5). mH2a/2m-3′B pre-mRNA contains an improved HDE that forms with the U7 snRNA an uninterrupted duplex of 16 base pairs and processing of this pre-mRNA in mouse nuclear extracts is SLBP-independent, with pre-binding of endogenous SLBP by excess stem-loop RNA (SL) or adding recombinant SLBP having no inhibitory or stimulatory effect, respectively (Figure 3F, compare lane 1 with lanes 3 and 4). The stimulatory effect of SLBP was even more striking when smaller amounts of the UV-eluted holo U7 snRNP were used in the reaction (1% of the sample compared to 5% in Figure 3E), with processing efficiency shifting from a barely detectable level in the absence of SLBP to as much as 50% in its presence (Figure 3G, lanes 2 and 3). SLBP mutants containing amino acid substitutions in α helix B of the RNA Binding Domain (Mut 1) or in the C-terminal region (Mut 2), that fail to interact with native U7 snRNP (34), also failed to enhance processing activity of the purified semi-recombinant holo U7 snRNP (Figure 3G, lanes 4 and 5). Overall, these results demonstrate that the purified semi-recombinant holo U7 snRNP is catalytically active, accurate in selecting the cleavage site and capable of cooperating with SLBP in achieving maximum efficiency of processing.

It was possible that the unexpected dependence of the semi-recombinant holo U7 snRNP on SLBP in cleaving mH2a/2m-3′B pre-mRNA resulted from deleting the entire unstructured C-terminal region from SmB. Our previous study suggested that SmB, SmD3 and Lsm10 likely use their C-terminal regions to bind histone pre-mRNA upstream of the HDE, stabilizing the complex between the substrate and the U7 snRNP (53). We used the UV-elution method to purify semi-recombinant holo U7 snRNP containing full length Lsm11 and SmB (Supplementary Figure S1A) but observed no reduction in the dependence of processing on SLBP (Supplementary Figure S1B, lanes 4 and 5, Figure S1C, lanes 2 and 3). Note that all ring proteins in this U7 snRNP were full length, resembling endogenous U7 snRNP. We also assembled core U7 snRNP in which Lsm11 lacked the loop sequence and SmB was truncated at the C-terminus. Semi-recombinant holo U7 snRNP reconstituted with these Lsm11 and SmB deletion mutants was active in processing and depended on SLBP for maximum activity (Supplementary Figure S2C, lanes 3 and 4). Clearly, the deleted regions of Lsm11 and SmB play no essential role in processing *in vitro*, although they are likely important *in vivo* (see Discussion). Unless otherwise indicated, core U7 snRNPs used in all subsequent experiments were assembled using these Lsm11 and SmB deletion variants. Note that with this approach, other parts of the Sm proteins, including structural and conserved elements in Lsm10 and Lsm11 (63), can be readily scanned for their potential roles in processing.

We took advantage of the reconstituted system to test whether processing activity of UV-eluted semi-recombinant holo U7 snRNP and its SLBP dependence can be affected by hnRNP Q or CstF64. hnRNP Q is one of relatively abundant RNA binding proteins of the mouse nuclear extract that we identified by mass spectrometry in a complex with the 3′ end region of H2a histone pre-mRNA (15,34,53). In the reconstituted system, this protein and other potential auxiliary factors are absent, providing a plausible explanation for the strong dependence of the UV-eluted U7 snRNP on SLBP. CstF64 plays an unclear role in processing of histone pre-mRNAs but it may be under-represented relative to the other subunits of endogenous HCC (15,34), resulting in a reduced processing activity of the purified semi-recombinant holo U7 snRNP in the absence of SLBP. We expressed hnRNP Q and CstF64 in bacteria (Supplementary Figure S2A and B) but observed that their addition to the reconstituted system either alone or together with SLBP had no detectable effect on processing activity (Supplementary Figure S2C, lanes 5–8). These results suggest that hnRNP Q plays no role in 3′ end processing of histone pre-mRNAs. Further studies with fully recombinant U7 snRNP will be required to conclude that CstF64 is a dispensable component of the HCC.

### Altering substrate specificity of semi-recombinant holo U7 snRNP

**Figure 4.**
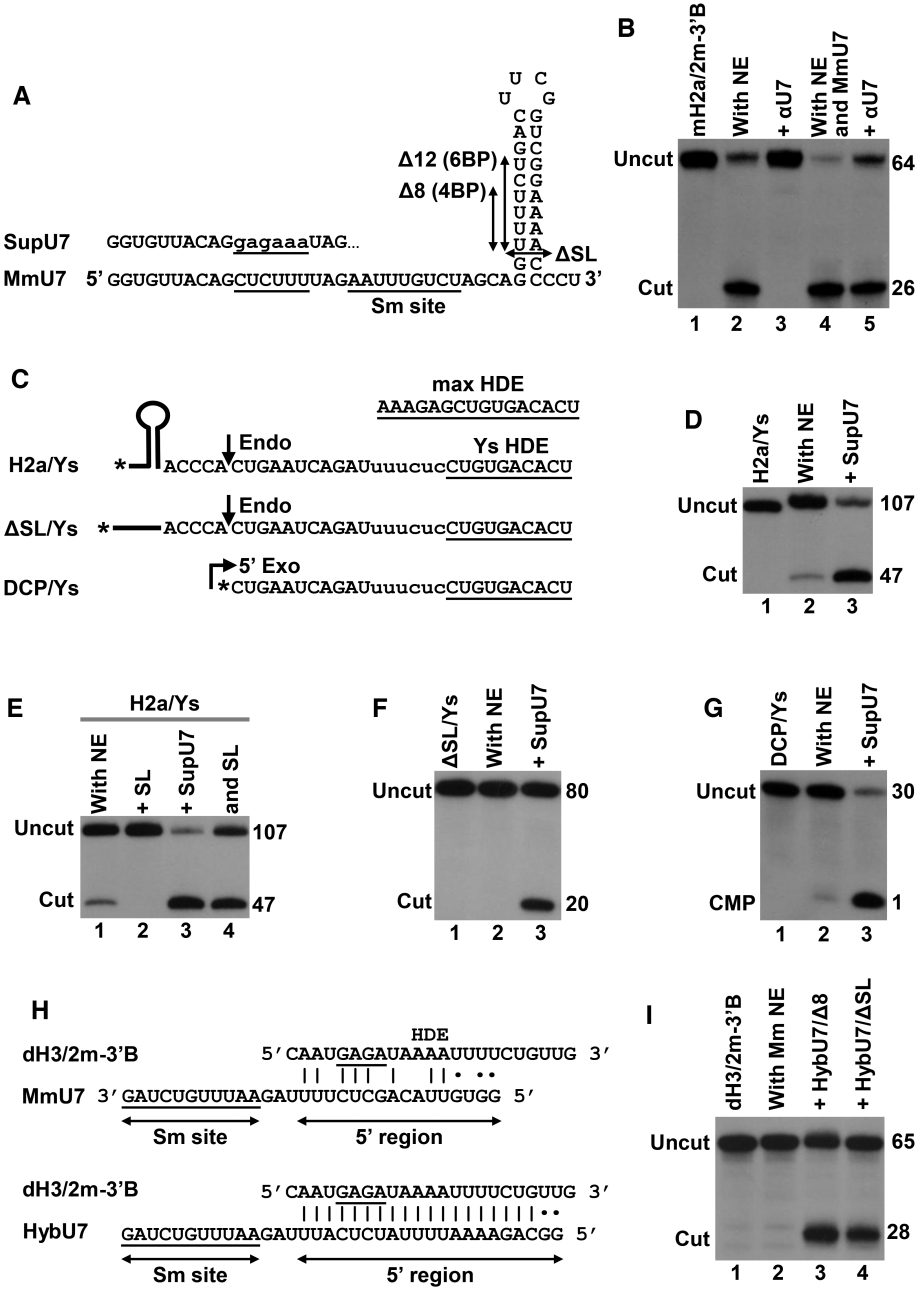
Altering substrate specificity of semi-recombinant holo U7 snRNP. (**A**) Sequence and structural organization of mouse U7 snRNA. The CUCUUU pyrimidine core of mouse (Mm) U7 snRNA and the nucleotide substitution (lower case letters) in the suppressor U7 snRNA (SupU7) are underlined. Deletions within the 3′ terminal stem-loop are indicated with double-headed arrows. (**B**) Processing of mH2a/2m-3′B pre-mRNA in a mouse nuclear extract (NE) alone or in the presence of an oligonucleotide blocking U7 snRNA (αU7) and/or recombinant core MmU7 snRNP. Lane 1 contains mH2a/2m-3′B pre-mRNA alone. (**C**) Changing six purines in an improved HDE of H2a histone pre-mRNA (max HDE) to six complementary pyrimidines (Ys, indicated with lower case letters) reduces the number of base pairs formed with endogenous U7 snRNA from 16 to 10 (underlined in the sequence). The three RNA substrates share the same mutated HDE but differ at 5′ end, with H2a/Ys containing normal stem-loop, ΔSL containing a 20-nucleotide unstructured region instead of the stem-loop, and DCP/Ys beginning immediately downstream of the cleavage site. Asterisks indicate the position of ^32^P label. (**D**–**G**) Processing of the mutant RNA substrates shown in panel C in a mouse nuclear extract (NE) alone or in the presence of SupU7 snRNP. (**H**). Base pairing potential between *Drosophila*-specific dH3/2m-3′B pre-mRNA and MmU7 or HybU7 snRNAs. Watson-Crick and G-U base pairs are represented by vertical lines and dots, respectively. The purine core of *Drosophila* HDE is underlined. (**I**). Processing of dH3/2m-3′B pre-mRNA in a Mm NE alone or in the presence of indicated semi-recombinant U7 snRNPs.

In addition to using chemically synthesized U7 snRNAs, we tested the functionality of U7 snRNAs generated by T7 transcription from linearized plasmids or duplex oligonucleotides. Using this approach, we initially generated wild type mouse U7 snRNA (MmU7) that besides containing a small number of additional nucleotides at the 5′ and 3′ ends had the same sequence as endogenous mouse U7 snRNA (Figure 4A). T7-generated MmU7 snRNA was used to assemble core U7 snRNP, bound to Δ51 FLASH, purified by SEC and assayed for the ability to support processing. To eliminate the influence of endogenous U7 snRNP, the purified complex of MmU7 snRNP and Δ51 FLASH was pre-bound to the 5′-labeled mH2a/2m-3′B pre-mRNA and then added to a mouse nuclear extract containing αU7 oligonucleotide. As expected, the oligonucleotide inhibited processing of mH2a/2m-3′B pre-mRNA in the absence of recombinant core MmU7 snRNP (Figure 4B, compare lanes 2 and 3). Importantly, pre-binding of the substrate to MmU7 snRNP rescued processing activity, alleviating the inhibitory effect of the oligonucleotide (Figure 4B, compare lanes 4 and 5). We conclude that T7-generated U7 snRNA, in spite of containing additional template-encoded nucleotides at both ends, assembled into a semi-recombinant U7 snRNP that successfully substituted for endogenous U7 snRNP. We used T7 transcription to generate U7 snRNAs in which 4 or 6 base pairs of the stem, or the entire 3′ terminal stem-loop, were deleted (Figure 4A, Δ8, Δ12 or ΔSL, respectively). These truncated U7 snRNAs reconstituted active semi-recombinant holo U7 snRNP in the mouse nuclear extract (see below), indicating that the 3′ stem-loop of U7 snRNA is not essential for the *in vitro* processing activity of U7 snRNP.

Mutations within the HDE that weaken base pairing with the U7 snRNA drastically reduce or inhibit processing (37,64,65). We generated H2a/Ys pre-mRNA by replacing the AAAGAG purine core in 107-nucleotide H2a/max pre-mRNA with UUUCUC, lowering the number of uninterrupted base pairs that are formed with endogenous U7 snRNA from 15 to 9 (Figure 4C). As a result of this mutation, H2a/Ys pre-mRNA was only weakly processed in a mouse nuclear extract, yielding less than 5% of the cleavage product during 60 min incubation at 32°C (Figure 4D, lane 2). In parallel, we generated suppressor (Sup) U7 snRNA in which the CUCUUU sequence that base pairs with the purine core of the HDE was replaced with GAGAAA (Figure 4A). This compensatory mutation restores the 15-base pair duplex with H2a/Ys pre-mRNA. A fraction of the SEC-purified complex of core SupU7 snRNP and Δ51 FLASH added to the nuclear extract strongly stimulated processing of H2a/Ys pre-mRNAs, increasing the amount of the cleavage product to ∼80% (Figure 4D, lane 3). This result clearly demonstrates that HDE mutations are efficiently suppressed by semi-recombinant holo U7 snRNP assembled on *in vitro* generated U7 snRNA. Note that in these and subsequent experiments the influence of endogenous U7 snRNP was nearly eliminated. As a result, the recombinant core U7 snRNPs can be added to the nuclear extract together with the mutant substrate (no pre-binding required) to form holo U7 snRNP and carry out the cleavage reaction.

The residual processing of H2a/Ys pre-mRNA in the mouse nuclear extract is likely due to the presence of SLBP, which stabilizes binding of endogenous U7 snRNP to the mutated HDE in H2a/Ys pre-mRNA (Figure 4D, lane 2, and Figure 4E, lane 1). Consistently, no processing of H2a/Ys pre-mRNA was observed in the presence of excess SL RNA that sequesters free SLBP in the extract (Figure 4E, lane 2). The same amount of SL RNA only partially inhibited processing observed in the presence of recombinant core SupU7 snRNP, by selectively eliminating the activity contributed by endogenous U7 snRNP (Figure 4E, compare lanes 2 and 3). The remaining activity contributed by SupU7 snRNP, which forms a 15-base pair duplex with H2a/Ys pre-mRNA, was SLBP-independent. This result argues against the possibility that semi-recombinant holo U7 snRNPs are intrinsically defective and unable to efficiently function without SLBP, as suggested by the behavior of the UV-eluted U7 snRNP incubated with substrate in the absence of any other protein.

We deleted the stem–loop from H2a/Ys pre-mRNA, generating ΔSL/Ys pre-mRNA (Figure 4C). Instead of the stem-loop, ΔSL/Ys pre-mRNA contains an unstructured sequence of 20 nucleotides, with the ACCCA sequence that precedes the cleavage site being unchanged. This substrate does not bind SLBP and in contrast to H2a/Ys is not cleaved by endogenous U7 snRNP (Figure 4F, lane 2). Processing was rescued in the presence of the core SupU7 snRNP/Δ51 FLASH complex (Figure 4F, lane 3), further confirming that semi-recombinant U7 snRNP with a strong complementarity to the HDE when tested in the extract rather than in a reconstituted processing reaction is capable of efficiently cleaving histone pre-mRNA in the absence of SLBP.

In addition to endonucleolytically cleaving histone pre-mRNAs, U7 snRNP also catalyzes 5′-3′ exonucleolytic degradation of the downstream cleavage product (DCP), releasing U7 snRNA from the base pairing interaction with the HDE for the next round of processing (23,39). Both enzymatic activities are provided by CPSF73 (23,66). We tested 5′-3′ exonucleolytic activity of SupU7 snRNP using DCP/Ys RNA, a substrate that corresponds to the DCP of H2a/Ys and ΔSL/Ys pre-mRNAs (Figure 4C). A 60-min incubation of the 30-nucleotide DCP/Ys in the mouse nuclear extract yielded only a small amount of labeled CMP (Figure 4G, lane 2), an indication of a low level of 5′-3′ degradation by endogenous U7 snRNP. In the presence of semi-recombinant holo SupU7 snRNP, the degradation efficiency reached 90% (Figure 4G, lane 3). We also tested the activity of semi-recombinant U7 snRNPs assembled on SupU7/Δ8 and SupU7/Δ12 snRNAs lacking 4 and 6 base pairs in the 3′ terminal stem, respectively. The two reconstituted semi-recombinant holo U7 snRNPs promoted efficient degradation of DCP/Ys RNA in a mouse nuclear extract (Supplementary Figure S3).

The HDEs of all five *Drosophila* histone pre-mRNAs are AU-rich and have a limited potential to base pair with mammalian U7 snRNAs (51,55). dH3/2m-3′B pre-mRNA contains the HDE from *Drosophila* histone H3 pre-mRNA (14,34) and may form with mouse U7 snRNA eleven, mostly AU and GU, base pairs that are interrupted by a number of mismatches (Figure 4H). As a result of this poor complementarity, dH3/2m-3′B pre-mRNA is not cleaved in mouse nuclear extracts (Figure 4I, lane 2). We tested whether this deficiency can be reversed by replacing the 5′ region in mouse U7/Δ8 and U7/Δ12 snRNAs (Figure 4A) with a 19-nucleotide sequence complementary to the HDE of dH3/2m-3′B pre-mRNA (Figure 4H). Including the two template-encoded guanosines added during T7 transcription, the 5′ end of the two new snRNAs, HybU7/Δ8 and HybU7/Δ12, forms 21 continuous base pairs with dH3/2m-3′B pre-mRNA (Figure 4H). Importantly, core HybU7/Δ8 and HybU7/ΔSL snRNPs complexed with Δ51 FLASH and added to a mouse nuclear extract where it bound the HCC efficiently cleaved the substrate during a 60 min incubation at 32°C (Figure 4I, lanes 3 and 4).

As expected, core HybU7/ΔSL snRNP cleaved dH3/2m-3′B pre-mRNA only when added to a mouse nuclear extract; in *Drosophila* Kc nuclear extract it strongly inhibited processing (Supplementary Figure S4A, lane 2). This dominant negative effect is likely a result of the efficient base pairing of the HybU7/Δ8 snRNA to dH3/2m-3′B pre-mRNA and the inability of mammalian Lsm11 and FLASH to recruit *Drosophila* HCC required for forming a productive semi-recombinant holo U7 snRNP.

The semi-recombinant holo U7 snRNP assembled in a mouse nuclear extract on U7 snRNA containing the entire sequence of *Drosophila* U7 snRNA (DmU7 snRNA) also cleaved dH3/2m-3′B pre-mRNA, although the activity was relatively low (Supplementary Figure S4B, lane 4). The 71-nucleotide *Drosophila* U7 snRNA is ∼10 nucleotides longer than other known U7 snRNAs and contains uridine rather than adenosine near the end of the Sm site (67). Previous studies in *Xenopus* oocytes suggested this adenosine may be important for the function of U7 snRNP in vertebrates (68), potentially explaining the weak activity of DmU7 snRNP in the mouse nuclear extract. Interestingly, this semi-recombinant holo U7 snRNP cleaved dH3/2m-3′B pre-mRNA 5 nucleotides after the stem-loop, thus functionally resembling endogenous mouse U7 snRNP rather than *Drosophila* U7 snRNP, which cleaves 4 nucleotides after the stem-loop (Supplementary Figure S4B, lanes 3–5). Thus, the strong tendency of the *Drosophila* processing machinery to cleave histone pre-mRNAs closer to the stem-loop is not dictated by particular features of the *Drosophila* U7 snRNA but likely results from the absolute dependence of *Drosophila* U7 snRNP on SLBP and other aspects of the *Drosophila* processing complex (34,51).

### The role of FLASH in endonucleolytic cleavage and 5′-3′ exonucleolytic degradation by U7 snRNP

**Figure 5.**
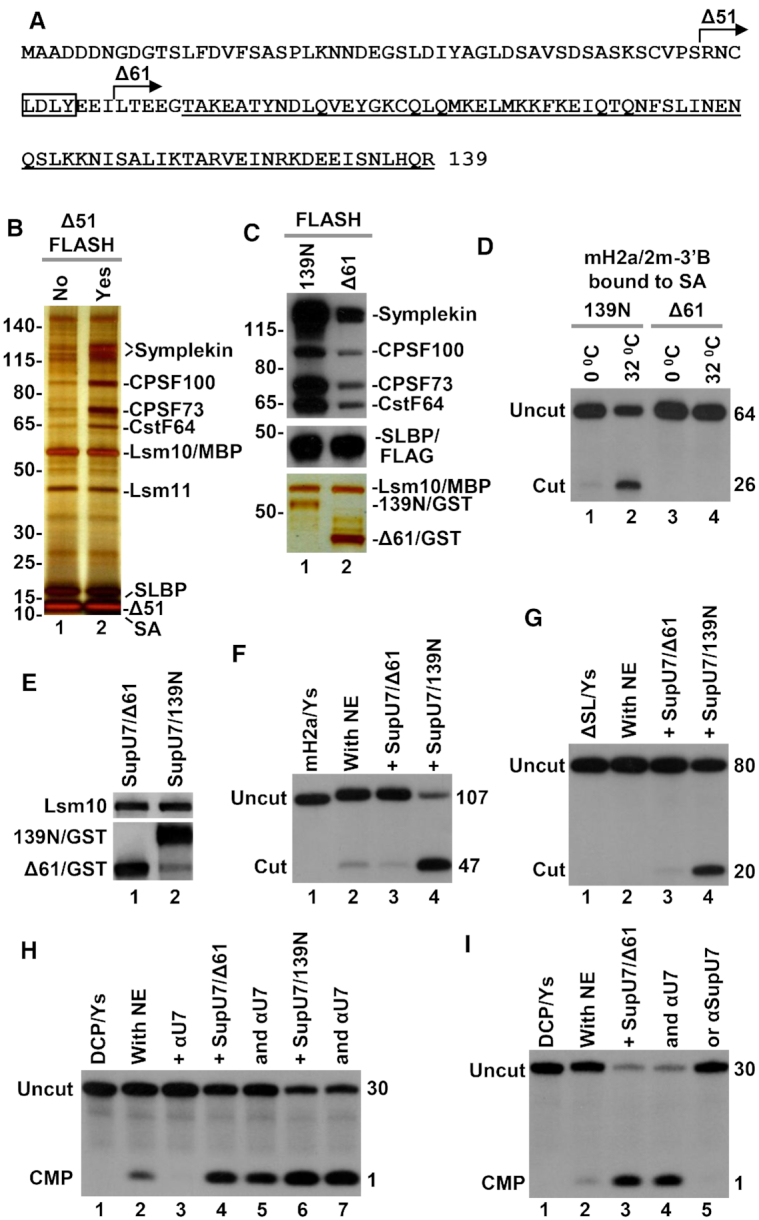
Role of FLASH in recruiting polyadenylation factors of the HCC to semi-recombinant holo U7 snRNP and its catalytic activity. (**A**) Amino acid sequence of the N-terminal region of FLASH. The essential LDLY motif is boxed and the start points of the Δ51 and Δ61 deletions are indicated with the arrows. The region that binds Lsm11 is underlined. (**B**) Proteins of mouse nuclear extract that associate with the *in vitro* assembled core U7 snRNP either lacking (lane 1) or containing (lane 2) recombinant Δ51 FLASH. The complexes were immobilized on SA beads via mH2a/2m-3′B pre-mRNA, as described in the legend for Figure 2A, and bound proteins were visualized by silver staining. (**C**) *In vitro* assembled core U7 snRNP was bound to either 139N FLASH or its inactive deletion mutant lacking the LDLY motif, Δ61. The resultant complexes were next bound in the presence of FLAG-tagged SLBP to radioactively labeled mH2a/2m-3′B pre-mRNA, immobilized on SA beads and incubated with mouse nuclear extract to reconstitute semi-recombinant holo U7 snRNP. The presence of the HCC components (symplekin, CPSF100, CPSF73 and CstF64), SLBP and each of the two forms of FLASH, each N-terminally tagged with GST, was monitored by Western blotting (top panel) and silver staining (bottom panel). (**D**) Small aliquots of immobilized processing complexes containing either 139N or Δ61 FLASH were incubated at 0°C and 32°C to evaluate their ability to support endonucleolytic cleavage of 5′-labeled mH2a/2m-3′B pre-mRNA. (**E–I**) *In vitro* assembled core SupU7 snRNP was bound to Δ61 or 139N FLASH. The complexes were purified by size exclusion chromatography, analyzed by Western blotting (panel E) and tested for the ability to support processing of H2a/Ys (panel F), ΔSL/Ys (panel G) and DCP/Ys (panels H and I) in a mouse nuclear extract.

A key role in stably recruiting the HCC by the FLASH/Lsm11 complex is played by the LDLY motif located in FLASH between amino acids 55–58 (13,56) (Figure 5A). FLASH mutants lacking this motif are inactive in endonucleolytic cleavage, converting U7 snRNP into a dominant negative factor that binds to histone pre-mRNA via U7 snRNA but fails to recruit the HCC, including the CPSF73 endonuclease (56). Surprisingly, this dominant negative effect was not observed in the 5′-3′ degradation assay, suggesting that in the absence of functional FLASH the HCC can transiently associate with U7 snRNP to act as a 5′-3′ exonuclease but not as an endonuclease (56). Now, with the availability of recombinant core U7 snRNP that can be associated *in vitro* with FLASH of choice, we reinvestigated this surprising difference between the two reactions in a more controlled way.

We first analyzed the role of FLASH in reconstitution of semi-recombinant holo U7 snRNP by immobilizing processing complexes on SA beads via mH2a/2m-3′B pre-mRNA, as illustrated in Figure 1A. Based on both silver staining (Figure 5B, lane 2) and mass spectrometry (not shown), pre-binding of recombinant core U7 snRNP to Δ51 FLASH resulted in an efficient recruitment of symplekin, CPSF100, CPSF73 and CstF64 to the complex. Only a small amount of these HCC subunits associated with the core U7 snRNP in the absence of Δ51 FLASH, likely as a result of recruiting endogenous FLASH (Figure 5B, lane 1). This result is consistent with our previous results showing that FLASH containing the LDLY motif is essential for a stable recruitment of the HCC (13).

We assayed immobilized complexes containing either functional (with the LDLY motif) or deficient FLASH (lacking the LDLY motif) for processing activity. mH2a/2m-3′B substrate labeled at the 5′ end with ^32^P was pre-bound in the presence of FLAG-tagged SLBP to the *in vitro* assembled core U7 snRNP containing either the first 139 amino acids of FLASH (139N FLASH) or Δ61 FLASH (amino acids 62–139), each N-terminally tagged with GST (Figure 5A). SLBP was used to increase the efficiency of complex formation, with the FLAG tag serving to monitor the amount of each purified complex by Western blotting. The pre-mRNA/SLBP/core U7/FLASH complexes were immobilized on SA beads, incubated with a nuclear extract to bind the HCC, and analyzed by Western blotting and silver staining. As expected, 139N FLASH in contrast to the functionally incompetent Δ61 FLASH promoted efficient assembly of the holo U7 snRNP (Figure 5C). The amount of SLBP was comparable in both complexes and Δ61 FLASH was even more abundant than 139N FLASH, clearly indicating that the poor binding of the HCC subunits in the presence of Δ61 FLASH resulted from deleting the LDLY motif, as previously shown (13,56).

A fraction of the beads was re-suspended in processing buffer and divided into small aliquots to test processing activity of the immobilized complexes. Semi-recombinant holo U7 snRNP assembled in the presence of 139N FLASH showed a trace of activity at 0°C and cleaved nearly 25% of the immobilized pre-mRNA at 32°C (Figure 5D, lanes 1 and 2, respectively). No activity at either temperature was detected for recombinant core U7 snRNP bound to Δ61 FLASH (Figure 5D, lanes 3 and 4). This experiment demonstrates that the ability of semi-recombinant holo U7 snRNP to endonucleolytically cleave histone pre-mRNA depends on the presence of a functional FLASH and stable recruitment of the HCC.

To confirm that the Δ61 deletion mutant of FLASH does not support the assembly of semi-recombinant holo U7 snRNP capable of endonucleolytic cleavage, we took advantage of Ys mutant substrates that are not recognized by endogenous U7 snRNP due to the presence of a purine-to-pyrimidine substitution within the HDE (Figure 4C). We assembled the Sm ring on SupU7/Δ8 snRNA and bound the resulting core U7 snRNP to GST-tagged 139N FLASH or Δ61 FLASH. Both complexes were purified by SEC and shown by Western blotting using anti-GST and anti-MBP antibodies to contain similar amounts of FLASH and Lsm10, respectively (Figure 5E). mH2a/Ys and ΔSL/Ys served as substrates to monitor the efficiency of endonucleolytic cleavage. DCP/Ys RNA was used to test the importance of FLASH in U7 snRNP-mediated 5′-3′ degradation of the downstream cleavage product.

mH2a/Ys pre-mRNA contains the stem-loop and was weakly cleaved by endogenous U7 snRNP due to the presence of SLBP in the extract (Figure 5F, lane 2), consistent with previous results (Figure 4D, lane 2). No cleavage during the same incubation time was observed for ΔSL/Ys substrate that lacks the stem-loop and does not bind SLBP (Figure 5G, lane 2), again in agreement with previous results (Figure 4F, lane 2). Core SupU7/Δ8 snRNP bound to the functionally deficient Δ61 FLASH showed no major effect on cleavage of mH2a/Ys (Figure 5F, lane 3) and ΔSL/Ys pre-mRNAs (Figure 5G, lane 3). Importantly, SupU7/Δ8 snRNP bound to 139N FLASH strongly stimulated cleavage of both substrates (lane 4 in Figure 5F and G).

Incubation of the 30-nucleotide DCP/Ys in the mouse extract yielded a small amount of labeled CMP (Figure 5H, lane 2). The release of the 5′ terminal nucleotide was blocked by the αU7 oligonucleotide (Figure 5H, lane 3), demonstrating that this weak degradation activity depends on endogenous U7 snRNP. The *in vitro* assembled SupU7/Δ8 snRNP bound to either Δ61 or 139N FLASH strongly stimulated degradation (Figure 5H, lanes 4 and 6) and this effect was not eliminated by αU7 oligonucleotide, consistent with most of the degradation being mediated by exogenous SupU7 snRNP (Figure 5H, lanes 5 and 7). Thus, Δ61 FLASH that fails to stably interact with the HCC and to promote endonucleolytic cleavage due to the absence of the LDLY motif is active in supporting 5′-3′ exonuclease activity of CPSF73.

To further confirm that the degradation is promoted by semi-recombinant holo SupU7/Δ8 snRNP, we used αSupU7, an antisense oligonucleotide complementary to the 5′ end of SupU7/Δ8 snRNA. In its presence, the release of labeled 5′ CMP by SupU7/Δ8 snRNP bound to Δ61 FLASH was inhibited (Figure 5I, line 5) and αU7 oligonucleotide that targets endogenous U7 snRNA and weakly base pairs with SupU7/Δ8 snRNA had no significant effect (Figure 5I, line 4). We conclude that in agreement with our previous experiments with endogenous U7 snRNP (56), binding of fully functional FLASH to Lsm11 is essential for endonucleolytic cleavage but dispensable for exonucleolytic degradation of the DCP.

### Length suppression by semi-recombinant holo U7 snRNP

In mammalian nuclear extracts, U7 snRNP cleaves histone pre-mRNAs at a fixed distance from the HDE (69). Based on this finding, we previously created mH2a+4 and mH2a+12 pre-mRNAs, two variants of mouse histone H2a (mH2a) pre-mRNA containing a 4- or 12-nucleotide insertion between the stem–loop and HDE (37). Compared to processing of WT H2a pre-mRNA, the cleavage site in these two substrates is shifted further away from the stem, extending the size of the upstream cleavage products by ∼4 and ∼12 nucleotides, respectively (37).

The shift in the position of the cleavage site in histone pre-mRNAs containing insertions between the SL and HDE can be reversed by expressing *in vivo* an altered U7 snRNA with similar size complementary insertions incorporated between the Sm binding site and the 5′ end region (70). We tested whether the same length suppression can be achieved *in vitro*. T7-generated U7 snRNAs containing various insertions or deletions between these two sequence elements were used to assemble recombinant core U7 snRNPs and analyzed for the ability to promote length suppression and cleavage site repositioning in mouse nuclear extracts.

**Figure 6.**
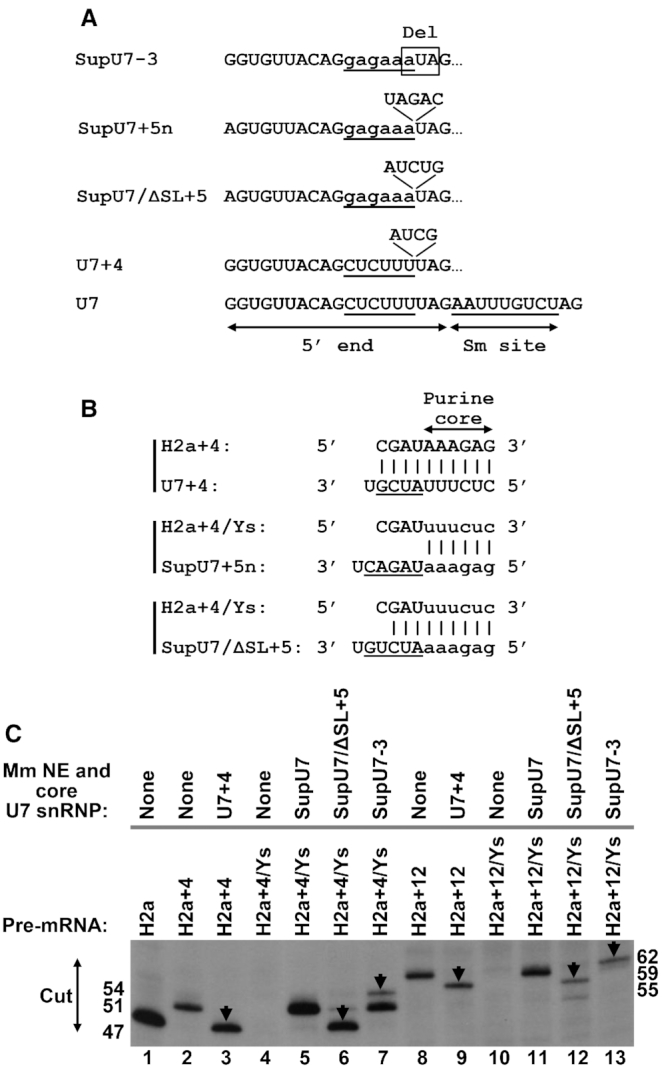
Length suppression by semi-recombinant holo U7 snRNP. (**A**) Insertions and a deletion in U7 snRNA tested for the ability to reposition the cleavage site during processing of histone pre-mRNAs. (**B**) Base pairing between nucleotides inserted in U7 snRNA and nucleotides of histone pre-mRNA located immediately upstream of the AAAGAG purine core (H2a+4) or its mutated variant UUUCUC (H2a+4/Ys). (**C**) *In vitro* processing of indicated pre-mRNAs in the presence of mouse nuclear extract and indicated recombinant core U7 snRNPs. Only the bottom portion of the gel containing the upstream cleavage products (cut) is shown. Products that result from shifting the cleavage site as a result of inserting or deleting nucleotides in U7 snRNA are indicated with the arrows.

In U7+4 snRNA, we inserted four nucleotides that base pair with the sequence in histone pre-mRNA located immediately 5′ of the purine core (Figure 6A and B). Otherwise, U7+4 snRNA had the same sequence as mouse U7 snRNA (Figure 6A). A SEC-purified complex of core U7+4 snRNP and Δ51 FLASH was pre-bound to mH2a+4 or mH2a+12 pre-mRNAs and incubated in a mouse nuclear extract to recruit the HCC. Note that pre-binding to the substrate was essential to eliminate the influence of endogenous U7 snRNA, which contains the same 5′ end region and hence the same substrate specificity as U7+4 snRNA. Processing of mH2a pre-mRNA in a mouse nuclear extract lacking recombinant core U7 snRNP generated an upstream products of 47 nucleotides and the product of mH2a+4 was extended by 4 nucleotides to 51 nucleotides, consistent with the molecular ruler mechanism (Figure 6C, compare lanes 1 and 2). Importantly, processing of mH2a+4 pre-mRNA pre-bound to *in vitro* assembled core U7+4 snRNP yielded a 47-nucleotide product, indicative of full length suppression (Figure 6C, lane 3). We conclude that *in vitro* assembled core U7 snRNP containing a 4-nucleotide insertion in its RNA component restores the correct position of the cleavage site in pre-mRNA substrate extended by the same number of nucleotides, consistent with previous *in vivo* studies (70). The same recombinant core snRNP also partially reversed the effect of the 12-nucleotide insertion, reducing the size of the product generated from mH2a+12 pre-mRNA by ∼4 nucleotides (Figure 6C, compare lanes 8 and 9).

To completely eliminate potential interference by endogenous U7 snRNP and the necessity of pre-binding of semi-recombinant U7 snRNPs and the substrates, we mutated the AAAGAG purine core in mH2a+4 and mH2a+12 to UUUCUC. The resultant mH2a+4/Ys and mH2a+12/Ys pre-mRNAs form with endogenous U7 snRNA only a weak duplex of 8 base pairs (including two GU base pairs) interrupted by one mismatch and are not cleaved in the mouse nuclear extract (Figure 6C, lanes 4 and 10, respectively). Importantly, cleavage was restored in the presence of core SupU7 snRNP containing a compensatory mutation in the 5′ end region that increases its base pairing interaction with the mutated HDE. As expected, processing of mH2a+4/Ys and mH2a+12/Ys pre-mRNAs by SupU7 snRNP yielded the same products as processing of mH2a+4 and mH2a+12 pre-mRNAs in the mouse extract by endogenous U7 snRNP (Figure 6C, compare lanes 2 and 5, and lanes 8 and 11).

We tested processing of mH2a+4/Ys and mH2a+12/Ys pre-mRNAs by two additional semi-recombinant U7 snRNPs, SupU7/ΔSL+5 and SupU7-3, containing either an insertion of five nucleotides or a deletion of 3 nucleotides between the Sm binding site and the 5′ region (Figure 6A). In SupU7/ΔSL+5 snRNA, 3 out of 5 added nucleotides base paired with the region inserted into histone pre-mRNAs (Figure 6B). Note that this chemically synthesized RNA was shortened by deleting the dispensable 3′ terminal stem-loop (ΔSL). The two U7 snRNPs behaved as expected, moving the cleavage site in mH2a+4/Ys and mH2a+12/Ys pre-mRNAs either closer (SupU7+5) or further away (SupU7-3) from the stem-loop, hence resulting in a shorter or a longer cleavage product, respectively (Figure 6C, lanes 6–7 and 12–13). SupU7-3 was only partially active in shifting the cleavage site in mH2a+4/Ys, with a relatively small amount of the upstream product being extended to 54 nucleotides (Figure 6C, lane 7). The majority of pre-mRNA was cleaved at the +4 site, yielding a 51-nucleotide product, indicative of no cleavage site repositioning. This might be explained by the contribution of SLBP, which holds U7 snRNP closer to the stem-loop hence, counterbalancing the effect of the deletion in the U7 snRNA, which in turn tends to move the cleavage site in the opposite direction, three nucleotides further downstream of the stem-loop. We also tested SupU7+5n snRNA that contains a 5-nucleotide insertion unable to form base pairs with the sequences inserted in mH2a+4/Ys and mH2a+12/Ys pre-mRNAs (Figure 6A and B). This recombinant core snRNP was inactive (not shown), consistent with the previous conclusion by Scharl and Steitz that effective length suppression requires that nucleotides inserted in the U7 snRNA are at least partially complementary to the pre-mRNA substrate (70).

### Core U7 snRNP assembled on a spliceosomal Sm binding site

**Figure 7.**
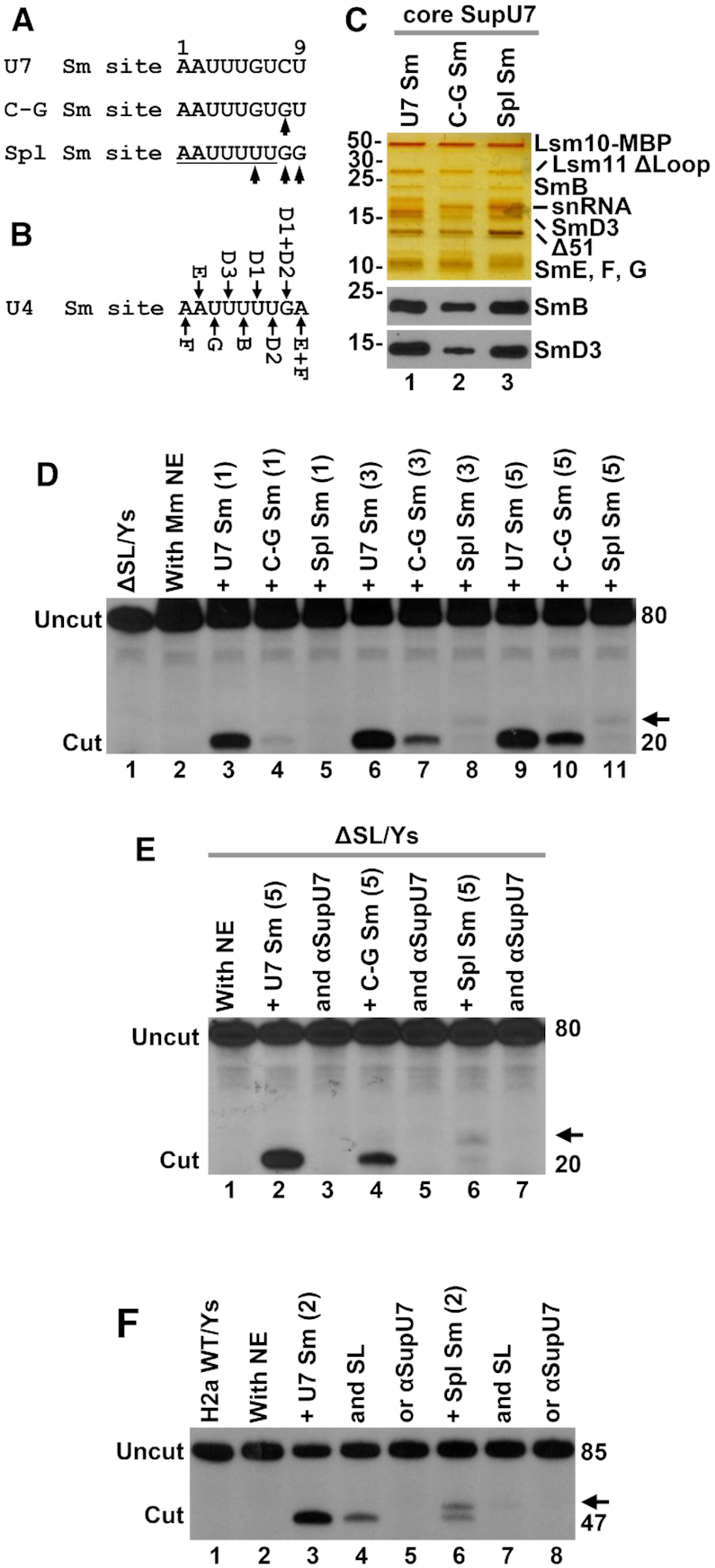
*In vitro* assembly of the U7-specific ring on a spliceosomal Sm site. (**A**) Nucleotide sequence of the U7-specific Sm site (top sequence) and changes made to generate C-G and spliceosomal-type (Spl) Sm sites (bottom sequences). Mutated nucleotides are indicated with the arrows. (**B**) Interaction between the spliceosomal Sm proteins and individual nucleotides of the Sm site in U4 snRNA (50). (**C**) Proteins of the U7-specific Sm ring bound to the U7, C-G and Spl Sm sites visualized by silver staining (top) and Western blotting (bottom). (**D**) Processing of ΔSL/Ys pre-mRNA in a mouse nuclear extract in the presence of 1, 3 and 5 μl of peak fraction containing indicated core U7 snRNPs. The arrow denotes the unusual cleavage product generated by SupU7 snRNP containing Spl Sm site. (**E**, **F**) Processing of ΔSL/Ys (panel E) and H2a WT/Ys (panel F) pre-mRNAs in a mouse nuclear extract in the presence of 5 or 2 μl of indicated core SupU7 snRNPs and processing competitors (SL or αSupU7).

The consensus sequence for the Sm binding site in U7 snRNA, AAUUUGUCU, differs in three positions (underlined) from the consensus AAUUUUUGG Sm binding site for the spliceosomal snRNAs (7,71,72) (Figure 7A). As shown *in vivo*, U7 snRNA containing a spliceosomal Sm site yielded an snRNP that was inactive in cleaving histone pre-mRNAs due to the lack of Lsm11 and Lsm10 that were replaced in the Sm ring by their spliceosomal counterparts, SmD1 and SmD2 (71,72).

We investigated whether T7-generated U7 snRNA with the spliceosomal AAUUUUUGG Sm binding site can support *in vitro* assembly of the U7-specific Sm ring containing Lsm10 and Lsm11. Previously, the assembly of the U7-specific Sm ring onto the spliceosomal Sm site was tested *in vivo*, i.e. in the presence of SmD1 and SmD2 whose intracellular concentrations highly exceed those for Lsm10 and Lsm11. In addition to replacing the U7 Sm binding site with the spliceosomal sequence, we also made a smaller change by substituting single cytosine at position 8 to guanosine (Figure 7A, C–G U7 snRNA). This cytosine and the following uridine (position 9) are invariant in U7 snRNAs (67) and are extremely rare in the spliceosomal snRNAs (7). They instead contain guanosine/uridine (position 8) and guanosine/adenine (position 9) that make cooperative interactions with SmD1/SmD2 and SmE/SmF, respectively (50) (Figure 7B). The changes within the Sm binding site were made in the context of suppressor U7 snRNA (SupU7) capable of recognizing Ys substrates containing the purine-to-pyrimidine substitution in the HDE (Figure 4C).

The seven recombinant proteins of the U7-specific Sm ring, including Lsm11 lacking the loop and SmB containing the C-terminal region (required for Western detection, see below), were incubated with wild type SupU7 snRNA, or its two variants containing substitutions within the Sm site, C–G Sm and Spl Sm (Figure 7A), and the assembled complexes were bound to Δ51 FLASH and purified by SEC. The elution profiles indicated that all three U7 snRNAs promoted the assembly of the U7-specific Sm ring (not shown). A fraction of each SEC-purified complex was analyzed by silver staining and showed to contain all Sm subunits of the ring (Figure 7C). We conclude that the spliceosomal-type Sm site can nucleate the assembly of a U7-specific Sm ring *in vitro*.

We tested 1, 2 and 5 μl of the peak SEC fraction (total 150 μl) of each core U7 snRNP/FLASH complex for the ability to support processing in a mouse nuclear extract. As the substrate, we initially used ΔSL/Ys pre-mRNA, which due to the lack of the stem-loop and mutations within the purine core of the HDE is not recognized by endogenous U7 snRNP, yielding no detectable cleavage product in a mouse nuclear extract (Figure 7D, lane 2). The addition of increasing amounts of core SupU7 snRNP containing the wild type Sm binding site (U7 Sm) to the nuclear extract resulted in an increasing efficiency of processing, with the highest amount used (5 μl) yielding ∼40% of the cleavage product during 60 min incubation (Figure 7D, lane 9). Recombinant core U7 snRNP with the C-G mutation supported correct processing, but its activity was weaker, with 5 μl of this mutant being less effective in generating the cleavage product than 1 μl of the wild type SupU7 snRNP (Figure 7D, compare lanes 3 and 10). This lower activity can be at least in part attributed to less efficient ring formation around the C-G Sm site (Figure 7C, lane 2). U7 snRNA with the Spl Sm site, in spite of efficiently promoting the ring assembly, gave rise to a semi-recombinant holo U7 snRNP that in a mouse nuclear extract was very inefficient in processing. Only a trace of the correct 20-nucleotide cleavage product was detected when 3 and 5 μl of this recombinant core U7 snRNP were added to the nuclear extract (Figure 7D, lanes 8 and 11). Interestingly, an additional slightly longer product was generated, suggesting that the U7-specific ring assembled on the spliceosomal Sm site misguides the CPSF73 endonuclease to a site located further away from the stem–loop. We tested a different SEC fraction eluted at the beginning of the peak and observed the same two cleavage products (data not shown). Both products were eliminated by an antisense oligonucleotide complementary to the 5′ end region of the SupU7 snRNA (Figure 7E, lane 7), as were the products generated by recombinant core U7 snRNPs containing wild type and C–G Sm sites (Figure 7E, lanes 3 and 5).

We tested whether the unusual cleavage pattern produced by recombinant core U7 snRNP containing Spl Sm site will be also observed with H2a WT/Ys pre-mRNA. In this substrate, the purine-to-pyrimidine mutation was made in the context of wild type H2a HDE rather than its improved version, as in case of H2a/Ys. As a result, H2a WT/Ys pre-mRNA in spite of containing the stem-loop and being able to interact with SLBP failed to stably recruit endogenous U7 snRNP and yielded no ‘background’ cleavage product in mouse nuclear extracts (Figure 7F, lane 2). Core SupU7 snRNP containing U7 Sm site efficiently cleaved H2a WT/Ys (Figure 7F, lane 3). Cleavage was inhibited in the presence of SL RNA, consistent with H2a WT/Ys pre-mRNA requiring SLBP to stably bind U7 snRNP (Figure 7F, lane 4), and by an antisense oligonucleotide complementary to the 5′ end of SupU7 snRNA (Figure 7F, lane 5). Core SupU7 snRNP containing a spliceosome-type Sm site (Spl Sm) was again only weakly active in processing, yielding two products of equal amount: the correct product and a new product longer by 3–4 nucleotides, with the generation of each being sensitive to the presence of the SL RNA and anti-U7 oligonucleotide (Figure 7F, lanes 6–8). These results strongly suggest that the Sm site in U7 snRNA is important for determining the site of cleavage in histone pre-mRNA.

## DISCUSSION

U7 snRNP is a multi-subunit endonuclease that cleaves replication-dependent histone pre-mRNAs 4–5 nucleotides downstream of a highly conserved stem-loop structure, generating mature histone mRNAs without a poly(A) tail (24). In the simplest core form, U7 snRNP consists of U7 snRNA and a heptameric ring containing Lsm10 and Lsm11 instead of the spliceosomal subunits SmD1 and D2 (6). Lsm11 binds FLASH (11), forming a platform for the recruitment of the Histone pre-mRNA Cleavage Complex (HCC), an assembly of 4 major polyadenylation factors: symplekin, CPSF100, CPSF73 and CstF64 (15), with CPSF73 acting as the 3′ endonuclease (23,38,73). We refer to this catalytically active form as holo U7 snRNP.

Detailed biochemical, structural and functional studies on endogenous holo U7 snRNP have been limited by its low concentration in animal cells (10,71). As a first step toward bypassing this limitation, we assembled core U7 snRNP using *in vitro* generated U7 snRNA and seven recombinant proteins of the U7-specific Sm ring. Analysis by electron microscopy revealed that assembled core U7 snRNP has a shape of a ring with an opening inside. A short tail attached to the ring is also visible and it likely represents the long 3′ terminal stem-loop of U7 snRNA. Overall, this image is highly reminiscent of the structure of core U1, U2, U4 and U5 snRNPs determined by electron microscopy and/or X-ray crystallography (46,48,74–76), strongly arguing that U7 snRNP in spite of containing two unique subunits, Lsm10 and Lsm11, and a unique Sm binding site in the U7 snRNA assembles into a similar heptameric donut-shaped core domain.

### Biochemical and functional analysis of semi-recombinant holo U7 snRNP

For biochemical and functional studies, recombinant core U7 snRNP bound to FLASH was incubated with a mouse nuclear extract to recruit the endogenous HCC. The semi-recombinant holo U7 snRNP reconstituted in this manner was active in processing and contained the same subset of polyadenylation factors that bind endogenous U7 snRNP: symplekin, CPSF100, CPSF73 and CstF64 (15,34). All these proteins were barely detectable if the *in vitro* assembled core U7 snRNP lacked recombinant FLASH, or was complexed with the Δ61 FLASH mutant that is unable to recruit the HCC due to the absence of the essential LDLY motif (amino acids 55–58). Altogether, these results provided evidence that the core U7 snRNP composed of recombinant U7 snRNA and Sm subunits gives rise to an active semi-recombinant holo U7 snRNP and confirmed our previous data that binding of the endogenous HCC to core U7 snRNP depends on the presence of a functional FLASH containing the LDLY motif.

U7 snRNP reconstituted in the presence of Δ61 FLASH (lacking the LDLY motif at positions 55–58), while being unable to stably recruit the HCC and endonucleolytically cleave histone pre-mRNAs, displayed a robust 5′-3′ exonuclease activity that degrades the downstream cleavage product. This result provides a strong argument that these two enzymatic activities of CPSF73 are mechanistically distinct and confirms our previous conclusion based on using a different experimental approach that stable binding of the HCC to the core U7 snRNP is critical for CPSF73 to act as an endonuclease but not as a 5′-3′ exonuclease (13,56).

To assemble core U7 snRNP, we used U7 snRNA that was either chemically synthesized or generated by T7 transcription, yielding in each case a catalytically active semi-recombinant holo U7 snRNP. Thus, the presence of additional nucleotides at the 5′ and 3′ ends of U7 snRNA that result from T7 transcription does not affect the activity of U7 snRNP. These results also argue that the function of U7 snRNA in processing does not depend on the presence of essential nucleotide modifications or the cap structure. Previous studies demonstrated that U2 snRNP reconstituted from T7-generated U2 snRNA failed to support *in vitro* splicing due to the absence of essential pseudouridine and 2′O-methyl modifications within the 5′ end region (77,78). In contrast, *in vitro* generated U5 snRNA, similarly to U7 snRNA, yielded a functional snRNP (77).

All known U7 snRNAs contain an extensive 3′ terminal stem-loop structure that is not conserved at the sequence level (4,79). Truncations of the stem from 11 to 6 base pairs or removal of the entire 3′ terminal stem-loop yielded functional U7 snRNP, indicating that this part of U7 snRNA has no direct role in processing. Previous studies demonstrated that U7 snRNAs lacking a stable stem at the 3′ end do not assemble into active U7 snRNPs in *Xenopusoocytes* (4,68). Clearly, the 3′ terminal stem-loop fulfills a number of essential functions *in vivo*, being important for 3′ end processing of the U7 snRNA precursor and transcriptional termination, stabilizing mature U7 snRNA against 3′ exonucleases and increasing the efficiency of Sm ring assembly via the SMN complex (44,80,81).

Of the seven recombinant proteins used to assemble active U7-specific Sm ring *in vitro*, SmE, SmF, SmG, SmB and SmD3 were expressed in bacteria. Thus, the function of these Sm subunits in processing of histone pre-mRNAs does not require essential post-translational modifications. Lsm10 and Lsm11 were expressed in insect cells using the baculovirus system. These two subunits are poorly conserved between vertebrates and invertebrates and hence are also unlikely to contain any modifications essential for processing. SmE, SmF, SmG, SmD3 and Lsm10 were full length. In some experiments, SmB lacked the entire unstructured C-terminal region (amino acids 96–324) and Lsm11 lacked amino acids 211–332 encompassing a large loop between the Sm1 and Sm2 motifs. Semi-recombinant holo U7 snRNPs reconstituted using these two truncated subunits were functionally indistinguishable from those that contained full length proteins. The deleted regions may be required for recruiting the SMN complex in the cytoplasm, other essential *in vivo* functions, or as in case of SmB, for splicing-related functions. These experiments illustrate the utility of our new system and how it can be used for mapping regions in the Sm ring that are essential for *in vitro* processing of histone pre-mRNAs.

Our studies demonstrated that the semi-recombinant holo U7 snRNP, when assayed in nuclear extracts, efficiently cleaves pre-mRNA substrates independently of SLBP, mimicking the *in vitro* behavior of endogenous U7 snRNP. Surprisingly, semi-recombinant holo U7 snRNP purified by UV-elution (15,62) and tested in a reconstituted reaction displayed a stronger dependence on SLBP in cleaving the same substrate. It is possible that nuclear extracts contain additional auxiliary factors that can partially substitute for the function of SLBP, facilitating binding of U7 snRNP to the HDE and/or presenting the substrate for cleavage. Based on our preliminary experiments, this function is unlikely to be mediated by hnRNP Q, a relatively abundant RNA binding protein frequently found to associate in mouse nuclear extracts with the 3′ end region of histone H2a pre-mRNAs (15,34,53). Further studies are required to test potential involvement of other candidate hnRNPs and their multiple splice variants in U7-dependent processing. It is also possible that the unexpected dependence of the reconstituted *in vitro* processing reaction on SLBP results from partial damage of semi-recombinant holo U7 snRNP during the UV-elution step, or other technical aspects of the method, rather than from the absence of an auxiliary factor.

### Changing substrate and cleavage specificities of semi-recombinant holo U7 snRNP

Substrate specificity of U7 snRNP is provided by the 5′ end region of U7 snRNA, which base pairs with the HDE of histone pre-mRNA. We tested whether mouse semi-recombinant holo U7 snRNP can cleave histone pre-mRNAs containing mutations within the HDE and whether the effect of these mutations can be suppressed by compensatory changes in U7 snRNA. Previously, the same approach was used *in vivo* to demonstrate that substrate recognition by U7 snRNP involves base pairing interaction between the 5′ end of U7 snRNA and the HDE (35,64,65). We replaced the AAAGAG purine core in the HDE of mH2a pre-mRNA with UUUCUC, reducing the number of base pairs formed with the mouse U7 snRNA and severely inhibiting processing in mouse nuclear extracts. Semi-recombinant SupU7 snRNP containing a compensatory mutation within the 5′ end of U7 snRNA (CUCUUU to AAAGAG) restored both the base pairing potential with the mutant pre-mRNA and its efficient processing. Collectively, these results clearly demonstrate that the substrate specificity of semi-recombinant U7 snRNP is determined by the sequence of the 5′ end region of the U7 snRNA component, mimicking the behavior of endogenous U7 snRNP.

Mouse nuclear extracts are unable to process *Drosophila* histone pre-mRNAs due to incompatibility of their HDEs with the mouse U7 snRNA. We tested whether mouse U7 snRNA can be reprogrammed to recognize *Drosophila*-specific histone pre-mRNAs in mouse nuclear extracts. By substituting the 5′ end region of mouse U7 snRNA with a sequence of 19 nucleotides complementary to the HDE of *Drosophila* histone H3 pre-mRNA, we assembled a core U7 snRNP that when added to a mouse nuclear extract reconstituted holo U7 snRNP capable of efficiently cleaving this pre-mRNA.

Cleavage of histone pre-mRNAs in mammalian nuclear extracts occurs at a fixed distance from the HDE, the site of U7 snRNP binding (69). Insertions that increase the spacing between the stem-loop and the HDE result in repositioning of the cleavage site to maintain the same distance from the HDE. This effect can be reversed by semi-recombinant holo U7 snRNP assembled on U7 snRNA containing a corresponding insertion between the Sm site and the 5′ end region. Thus, U7 snRNP reconstituted from both recombinant and endogenous components is also capable of length suppression, mimicking the activity of U7 snRNPs assembled *in vivo* (70). As previously concluded (70), our *in vitro* results indicate that the sequence inserted in the U7 snRNA of the *in vitro* reconstituted U7 snRNP must be at least partially complementary to the pre-mRNA. Consistent with the mechanism of length suppression, semi-recombinant U7 snRNP assembled on U7 snRNA with a short deletion between the Sm site and the 5′ end causes the opposite effect; the cleavage site moves further away from the stem–loop, enhancing rather than suppressing the effect of insertions in the pre-mRNA substrate. Altogether, our results indicate that by simply changing the sequence of the 5′ end region in U7 snRNA and its distance to the Sm binding site, the semi-recombinant U7 snRNP can be converted into an endoribonuclease of desired substrate and cleavage specificities.

### The U7-specific Sm binding site plays an important role in determining the cleavage site

The AAUUUUUGA sequence of the human U4 Sm site is sufficient to assemble seven spliceosomal Sm proteins into a heptameric Sm ring *in vitro* (46,82). According to recently re-refined structures, each of the first seven nucleotides of this sequence interacts with a single Sm subunit of the ring in the following register: SmF, SmE, SmG, SmD3, SmB, SmD1 and SmD2. The penultimate guanosine (position 8) and the terminal adenosine (position 9) are recognized co-operatively by SmD1 and SmD2, and SmE and SmF, respectively (50). The same mode of interaction is used by the seven spliceosomal Sm proteins to recognize the AAUUUGUGG Sm site from U1 snRNA, with the exception that SmD1 binds the guanosine at position 6 rather than uridine present in U4 snRNA (50).

The U7-specific Sm site, AAUUUGUCU, differs from the AAUUUUUGG spliceosomal consensus, with the terminal CU dinucleotide being virtually invariable in U7 snRNAs and never found at this position in the spliceosomal snRNAs (7). The *in vivo* expression of a mutant U7 snRNA containing the spliceosomal Sm site resulted in the incorporation of SmD1 and SmD2 instead of Lsm10 and Lsm11, yielding core U7 snRNP that failed to support processing of histone pre-mRNAs (9,72). *In vitro*, in the absence of the SmD1 and SmD2 proteins, the same mutant U7 snRNA promoted the assembly of a stable Sm ring containing Lsm10 and Lsm11, allowing for the first time functional studies on the resultant chimeric core U7 snRNP. Strikingly, this core U7 snRNP displayed only a weak processing activity when added to mouse nuclear extracts, inefficiently cleaving histone pre-mRNA at both the correct site and a new site located 3–4 nucleotides further downstream. Cleavage at this downstream site was never observed for endogenous U7 snRNP or the recombinant U7 snRNP containing the wild type Sm site. While the molecular basis for this altered cleavage activity is unknown, one possibility is that binding of Lsm10 and Lsm11 to the spliceosomal Sm site affects the overall geometry of the U7-specific Sm ring, resulting in misalignment of the CPSF73 endonuclease with the substrate. This possibility will be addressed by future structural studies that will take advantage of the successfully assembled and functionally competent core U7 snRNP described in this report.

## DATA AVAILABILITY

The mass spectrometry proteomics data have been deposited to the ProteomeXchange Consortium via the PRIDE (83) partner repository with the dataset identifier PXD014164.

## Supplementary Material

gkz1148_Supplemental_File

## Notes

Present address: Wei Shen Aik, Department of Chemistry, Hong Kong Baptist University, Kowloon Tong, Kowloon, Hong Kong SAR.

Present address: Kaituo Wang, Department of Biomedical Sciences, University of Copenhagen, Copenhagen, Denmark.
