# Supplementary material for: Composition and processing activity of a semi-recombinant holo U7 snRNP
Source: Nucleic Acids Res. 2019 Dec 10;48(3):1508–30. doi: 10.1093/nar/gkz1148 (PMC7026596; doi:10.1093/nar/gkz1148)
Supplement: gkz1148_Supplemental_File [file gkz1148_supplemental_file.pdf]

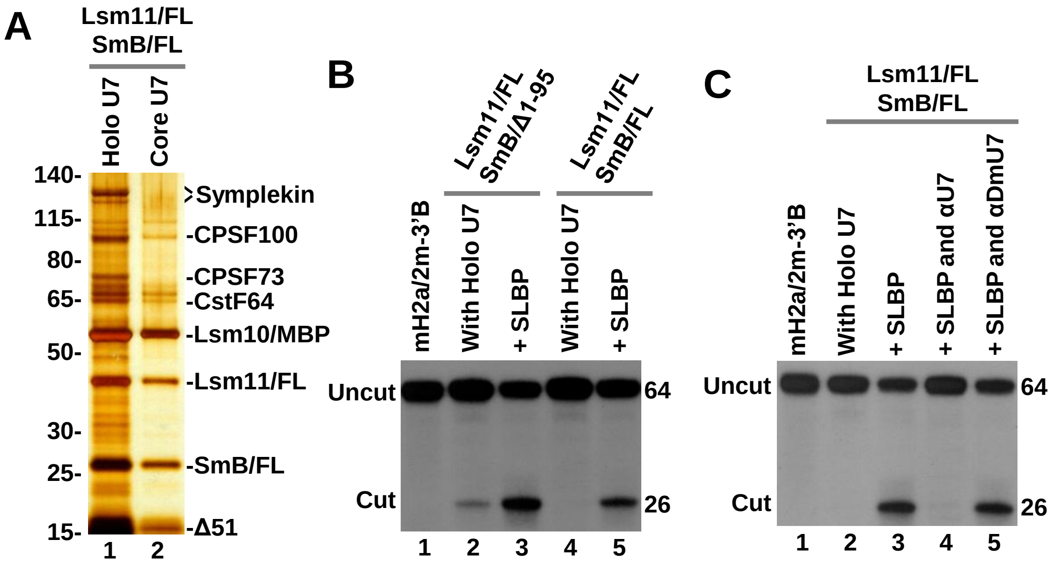

Figure S1. **Analysis of processing activity of UV-eluted holo U7 snRNP. A.** Semi-recombinant holo U7 snRNP and recombinant core U7 snRNP containing full length Lsm11 (Lsm11/FL) and SmB (SmB/FL) were assembled on U7-5'pcB snRNA and UV-eluted, as described in the text. A fraction (10%) of the UV-eluted material was separated by 4-12% SDS/polyacrylamide gel electrophoresis and analyzed for protein composition by silver staining. **B.** A fraction (2.5%) of the UV-eluted semi-recombinant holo U7 snRNP containing Lsm11/FL and SmB/FL was analyzed for cleavage activity in a reconstituted processing reaction either alone (lane 4) or in the presence of SLBP (lane 5). The same two reactions with the UV-eluted semi-recombinant holo U7 snRNP containing Lsm11/FL and SmB/Δ1-95 are shown for comparison in lanes 2 and 3, respectively. Lane 1 contains unprocessed mH2a/2m-3'B pre-mRNA. **C.** A fraction (2.5%) of UV-eluted semi-recombinant holo U7 snRNP containing Lsm11/FL and SmB/FL was analyzed for cleavage activity in a reconstituted processing reaction either alone (lane 2) or in the presence of SLBP and indicated oligonucleotides (lanes 3-5). αU7 and αDmU7 oligonucleotides are complementary to the 5' end of mouse/human and *Drosophila* U7 snRNAs, respectively. Note that αDmU7 serves as a negative control and does not block processing.

A

CstF64 (577 AA)

MAGLTVRDPAVDRSLRSVFVGNIPYEATEEQLKDIFSEVGPVVSFRLVYDRETGKPKGYGFC
EYQDQETALSAMRNLNGREFSGRALRVDNAASEKNKEELKSLGTGAPVIESPYGETISPEDA
PESISKAVASLPPEQMFELMKQMKLCVQNSPQEARNMLLQNPQLAYALLQAQVVMRIVDPEI
ALKILHRQTNIPTLIAGNPQPVHGAGPGSGSNVSMNQQNPQAPQAQSLGGMHVNGAPPLMQA
SMQGGVPAPGQMPAAVTGPGPGSLAPGGGMQAQVGMPGSGPVSMERGQVPMQDPRAAMQRGS
LPANVPTPRGLLGDAPNDPRGGTLLSVTGEVEPRGYLGPPHQGPPMHHVPGHESRGPPPHEL
RGGPLPEPRPLMAEPRGPMLDQRGPPLDGRGGRDPRGIDARGMEARAMEARGLDARGLEARA
MEARAMEARAMEARAMEARAMEVRGMEARGMDTRGPVPGPRGPIPSGMQGPSPINMGAVVPQ
GSRQVPVMQGTGMQGASIQGGSQPGGFSPGQNQVTPQDHEKAALIMQVLQLTADQIAMLPPE
QRQSILILKEQIQKSTGAP

hnRNP Q (527 AA)

MATEHVNGNGTEEPMDTTSAVIHSENFQTLLDAGLPQKVAEKLDEIYVAGLVAHSDLDERAI
EALKEFNEDGALAVLQQFKDSDLSHVQNKSAFLCGVMKTYRQREKQGTKVADSSKGPDEAKI
KALLERTGYTLDVTTGQRKYGGPPPDSVYSGQQPSVGTEIFVGKIPRDLFEDELVPLFEKAG
PIWDLRLMMDPLTGLNRGYAFVTFCTKEAAQEAVKLYNNHEIRSGKHIGVCISVANNRLFVG
SIPKSKTKEQILEEFSKVTEGLTDVILYHQPDDKKKNRGFCFLEYEDHKTAAQVKVLFVRNL
ANTVTEEILEKAFSQFGKLERVKKLKDYAFIHFDERDGAVKAMEEMNGKDLEGENIEIVFAK
PPDQKRKERKAQRQAAKNQMYDDYYYYGPPHMPPPTRGRGRGGRGGYGYPPDYYGYEDYYDY
YGYDYHNYRGGYEDPYYGYEDFQVGARGRGGRGARGAAPSRGRGAAPPRGRAGYSQRGGPGS
ARGVRGARGGAQQQRGRGQGKGVEAGPDLLQ

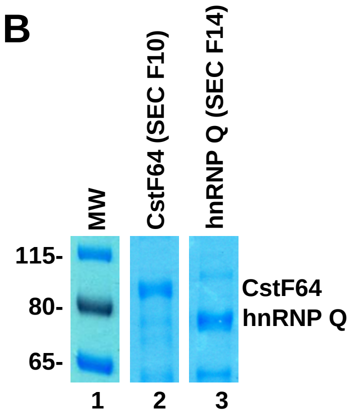

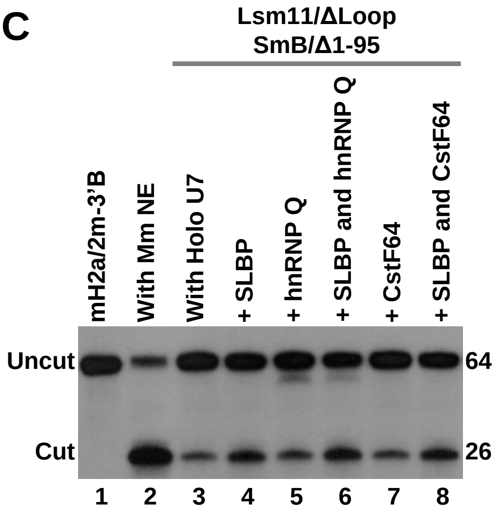

Figure S2. **Testing the role of CstF64 and hnRNP Q in processing. A.** Amino acid sequences of CstF64 and hnRNP Q splice variants used in the analysis. **B.** CstF64 and hnRNP Q with an N-terminal GST tag were expressed in bacteria and purified on nickel beads via the His tag followed by size exclusion chromatography (SEC). The peak SEC fractions containing purified CstF64 (fraction 10, F10) or hnRNP Q (fraction 14, F14) were separated by SDS-page and analyzed by Coomassie staining. **C.** A fraction (2.5%) of the UV-eluted semi-recombinant holo U7 snRNP containing Lsm11/ΔLoop and SmB/Δ1-95 was analyzed for cleavage activity in a reconstituted processing reaction either alone (lane 3) or in the presence of 5 pmol of indicated proteins (lanes 5-8). Lane 1 contains unprocessed mH2a/2m-3'B pre-mRNA. Processing of the substrate by mouse nuclear extract (NE) is shown in lane 2.

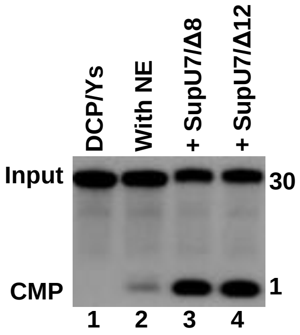

Figure S3. Degradation of ΔSL/Ys RNA by core SupU7 snRNP. ΔSL/Ys RNA substrate was incubated in a mouse nuclear extract (NE) alone or in the presence of core SupU7 snRNP that was assembled on U7 snRNA with either 4 or 6 bases pairs (Δ8 and Δ12, respectively) of the 3' terminal stem-loop being deleted. The degradation was measured as the release of the 5' terminal ^{32}P-labeled CMP during 60 min incubation at 32 °C. Input ΔSL/Ys RNA is shown in lane 1.

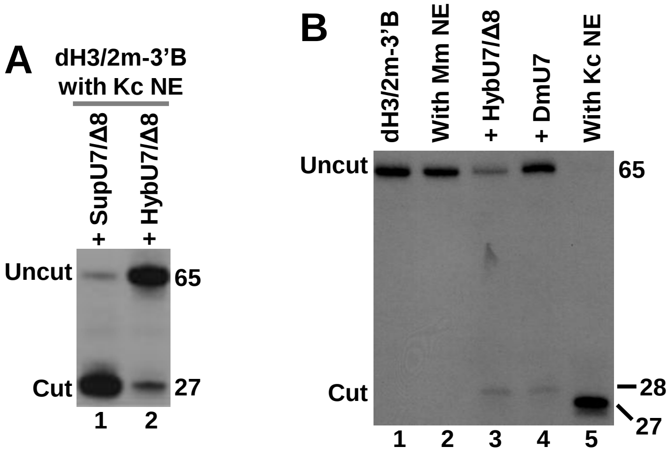

Figure S4. Processing of *Drosophila*-specific dH3/2m-3'B pre-mRNA in mouse and *Drosophila* nuclear extracts. A. Processing of dH3/2m-3'B pre-mRNA in *Drosophila* Kc nuclear extract containing SupU7/Δ8 (lane 1) or HybU7/Δ8 (lane 2) core snRNPs. Note that SupU7/Δ8 core U7 snRNP does not base-pair with dH3/2m-3'B pre-mRNA and has no inhibitory effect on processing of dH3/2m-3'B pre-mRNA. **B.** Processing of dH3/2m-3'B pre-mRNA in a mouse (Mm) NE alone (lane 2) or in the presence of following recombinant core U7 snRNPs: HybU7/Δ8 (lane 3) and Dm U7 (lane 4). The same pre-mRNA incubated in *Drosophila* Kc nuclear extract is shown in lane 5. Lane 1 contains unprocessed dH3/2m-3'B pre-mRNA.
